# Exploring the Mechanisms of Multiple Insecticide Resistance in a Highly *Plasmodium*-Infected Malaria Vector *Anopheles funestus* Sensu Stricto from Sahel of Northern Nigeria

**DOI:** 10.3390/genes11040454

**Published:** 2020-04-22

**Authors:** Sulaiman S. Ibrahim, Muhammad M. Mukhtar, Helen Irving, Jacob M. Riveron, Amen N. Fadel, Williams Tchapga, Jack Hearn, Abdullahi Muhammad, Faruk Sarkinfada, Charles S. Wondji

**Affiliations:** 1 Vector Biology Department, Liverpool School of Tropical Medicine (LSTM), Liverpool L3 5QA, UK; Helen.Irving@lstmed.ac.uk (H.I.); jack.hearn@lstmed.ac.uk (J.H.); Abdullahi.Muhammad@lstmed.ac.uk (A.M.); charles.wondji@lstmed.ac.uk (C.S.W.); 2 Department of Biochemistry, Bayero University, PMB 3011 Kano, Nigeria; muhammadmahemukhtar@gmail.com; 3 LSTM Research Unit, Centre for Research in Infectious Diseases (CRID), P.O. Box 13591 Yaoundé, Cameroon; Jacob.Riveron_Miranda@syngenta.com (J.M.R.); amen.fadel@crid-cam.net (A.N.F.); williams.tchapga@crid-cam.net (W.T.); 4 Centre for Biotechnology Research, Bayero University, PMB 3011 Kano, Nigeria; 5 Department of Medical Microbiology, Bayero University, PMB 3011 Kano, Nigeria; fsfada.mcp@buk.edu.ng

**Keywords:** *Anopheles funestus*, resistance, metabolic, 119F mutation, *GSTe2*, *Plasmodium falciparum*, malaria

## Abstract

The Nigerian Government is scaling up the distribution of insecticide-treated bed nets for malaria control, but the lack of surveillance data, especially in the Sudan/Sahel region of the country, may hinder targeting priority populations. Here, the vectorial role and insecticide resistance profile of a population of a major malaria vector *Anopheles funestus* sensu stricto from Sahel of Nigeria was characterised. *An. funestus* s.s. was the only vector found, with a high human blood index (100%) and a biting rate of 5.3/person/night. High *Plasmodium falciparum* infection was discovered (sporozoite rate = 54.55%). The population is resistant to permethrin (mortality = 48.30%, LT_50_ = 65.76 min), deltamethrin, DDT (dichlorodiphenyltrichloroethane) and bendiocarb, with mortalities of 29.44%, 56.34% and 54.05%, respectively. Cone-bioassays established loss of efficacy of the pyrethroid-only long-lasting insecticidal nets (LLINs); but 100% recovery of susceptibility was obtained for piperonylbutoxide (PBO)-containing PermaNet^®^3.0. Synergist bioassays with PBO and diethyl maleate recovered susceptibility, implicating CYP450s (permethrin mortality = 78.73%, χ^2^ = 22.33, *P* < 0.0001) and GSTs (DDT mortality = 81.44%, χ^2^ = 19.12, *P* < 0.0001). A high frequency of 119F *GSTe2* mutation (0.84) was observed (OR = 16, χ^2^ = 3.40, *P* = 0.05), suggesting the preeminent role of metabolic resistance. These findings highlight challenges associated with deployment of LLINs and indoor residual spraying (IRS) in Nigeria.

## 1. Introduction

Nigeria tops the list of the countries with the highest burden of malaria, accounting for ~25% of all malaria cases worldwide and 19% of the 435,000 malaria-related deaths [1]. Though the WHO African Region accounted for 88% of the 172,000 fewer global malaria deaths reported in 2017 compared to 2010 [1], the reduction in malaria cases stalled between 2015–2017 [1,2], due to lack of progress in countries like Nigeria, which reported increased cases of more than half a million in 2017, compared with 2016 [1]. With *Anopheles gambiae* sensu lato and *Anopheles funestus* sensu stricto (*An. funestus*) as the major malaria vectors, and *Plasmodium falciparum* as the major malaria-causing species (100% of all cases in 2017), it is not surprising that this disease accounts for ~60% of outpatient visits to health facilities and 30% of child mortality in Nigeria [3]. The lack of progress towards malaria pre-elimination in Nigeria is partly due to insufficient and/or discordant entomological and active case surveillance data [4], which are important guides to identify priority areas and the most vulnerable populations to implement data-driven decisions. In stark contrast to *An. gambiae* s.l. [5,6,7], the major malaria vector *An. funestus* from the Sudan/Sahel savannah of northern Nigeria has been neglected for decades, after comprehensive works conducted by several pioneers, before 1960. These almost seven decades old studies include (i) the work of Bruce-Chwatt and Haworth (carried out in 1955–56), which described *An. funestus* populations from Sokoto, north-western Nigeria, highly resistant to DDT (dichlorodiphenyltrichloroethane), dieldrin, and benzene hexachloride [8]; (ii) a detailed examination of *An. funestus* species, published in 1959 by W.M. Service [9] and its role in transmission in northern Nigeria [10]; as well as (iii) a 1964 small-scale hut trials to establish impact of DDT and malathion exposure on behaviour of *An. funestus* and *An. gambiae* [11]. After the Garki Project (1960–1970) [12], interest in *An. funestus* waned in northern Nigeria, though it is the chief vector in the dry season [13], extending the period of malaria transmission when densities of *An. gambiae* s.l. have declined [14]. Due to its high vectorial capacity, conferred by its unusually high anthropophilic and endophilic behaviour [14,15], this species is very important target, which should not be neglected if the ambitious target of the WHO to reduce global malaria case incidence by 90% is to be realised [16]. Contrary to the north, several studies have characterised *An. funestus* populations from southern Nigeria. For example, the role of this vector in malaria transmission was established in populations from four sites in southwest Nigeria [17], and recently its role in transmission and insecticide resistance profile was investigated by Djouaka and colleagues [18]. Unfortunately, information on this vector species from southern Nigeria cannot be extrapolated to the north, because Nigeria has five ecological zones which define intensity and seasonality of transmission and heterogeneity in mosquito vector compositions [19]. 

Here, a primary data from study of the major malaria vector *An. funestus* is presented. The role of this vector from Sahel of northern Nigeria in malaria transmission was investigated, and its resistance status to the major public health insecticides in use for bed nets and indoor-residual spraying established. The possible mechanisms driving metabolic resistance in the field were also investigated using the synergist bioassays and TaqMan genotyping for the 119F glutathione S-transferase (*GSTe2*) mutation, which confers resistance to DDT and pyrethroids [20]. The presence of the 1014F/S knockdown resistance (*kdr*) mutations previously associated with pyrethroid resistance [21,22], and the *acetylcholinesterase-1* (*ace-1*) G119S [23] and N485I [24] mutations associated with carbamate/organophosphate resistance, was also assessed through sequencing. Findings of this study could guide the Nigerian National Malaria Elimination Program to implement evidence-based resistance management and malaria control measures in northern Nigeria as it scales up distribution of insecticide treated net (ITNs) as part of the goals of the National Malaria Strategic Plan (NMSP) 2014–2020 [25].

## 2. Materials and Methods 

### 2.1. Study Site and Mosquito Sampling

**Figure 1:**
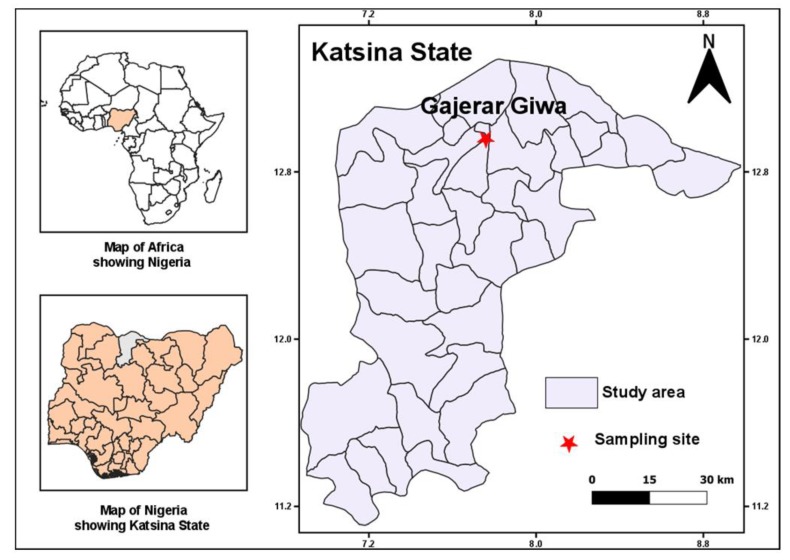
A map showing the sampling locality (Gajerar Giwa) in the Sahel of northern Nigeria.

Blood-fed female *Anopheles* mosquitoes resting indoors were collected between 17–20 November 2018 using battery-operated aspirators (John. W. Hock, Gainesville, FL, USA). Collection was done in eight randomly selected houses (among those who consented), in the morning hours (6:00–7:00 a.m.) at Gajerar Giwa (13°11′57.1″ N 7°45′53.5″ E), a village in Katsina State, north-western Nigeria. Located in the semi-arid savannah, Gajerar Giwa (Figure 1) neighbors Ajiwa Dam, built in 1975 and is used by nearby communities, for domestic purposes, fishing, and the year-round irrigation of vegetables, including tomato, lettuce, pepper, etc. Farmers apply quantities of pesticides mainly organophosphate-based, as well as pyrethroids and carbamates for the control of insect pests, undesirable herbs and fungi (http://documents.worldbank.org/curated/pt/244751486100486129/pdf/SFG2945-EA-P148616-Box402883B-PUBLIC-Disclosed-1-31-2017.pdf).

Clearance for indoor collection previously obtained from Operational Research Advisory Committee, Ministry of Health (with reference number MOH/off/797/TI/402) was used. The blood fed females obtained were maintained on 10% sugar at 25 °C ± 2 and 70–80% relative humidity for 6–7 d. Gravid females were transferred into 1.5 mL tubes individually and forced to lay eggs, using established protocols [26]. The F_0_ parents were identified as belonging to the *An. funestus* group using morphological keys [27] and confirmed as *An. funestus* s.s. using the cocktail polymerase chain reaction PCR [28]. Egg batches were transferred into paper cups for hatching in the insectary located at Bayero University Kano, Nigeria. Eggs that hatched were pooled into bowls and supplemented with Tetramin^TM^ baby fish food. The 3- to 4-days old F_1_ females that emerged were mixed in cages and used for bioassays. DNA extracted from all F_0_ and F_1_ females and to be used for molecular analyses were transported to the Liverpool School of Tropical Medicine (LSTM), UK, under the DEFRA license (PATH/125/2012, UK). To establish the indoor resting densities of the *An. funestus* and the source of blood meal, a pyrethrum spray collection was conducted indoor in the eight houses on 21 November 2018. 

### 2.2. Molecular Identification of Mosquito Species

Genomic DNA (gDNA) was extracted from the females which laid eggs, using the Livak protocol [29], and the mosquitoes identified to species using the cocktail PCR of Koekemoer, et al. [28]. These include all the females from the first 3 days of collections, as well as all the females collected on day 4, which were used for the estimation of indoor resting density and identification of blood meal source. 

### 2.3. Estimation of Entomological and Parasitological Parameters

#### 2.3.1. Estimation of Indoor Resting Density, Human Blood Index and Biting Rate

The indoor resting density (IRD) was calculated from the number of *An. funestus* collected on the 4th d, relative to the number of houses assessed, as advised by the WHO [30,31]. A total of 112 blood-fed females were dissected <48 h after collection, separating heads/thoraces from the abdomens. The abdomens were used for gDNA extraction as outlined in WHO guidelines [30] using the DNeasy Blood and Tissue Kit (QIAGEN, Hilden, Germany), according to manufacturer’s protocol. Identification of the source of blood was conducted using the cocktail PCR of Kent et al. [32]. The human blood index was calculated from the number of females that have fed on humans, relative to the total number of females caught [30,31]. The human biting rate was estimated from the number of the females that have fed on humans, relative to the total number of people in the houses sampled. 

#### 2.3.2. Estimation of Sporozoite Rate

Sixty-six females (22 individuals randomly selected from each of the three days of indoor collection) which laid eggs successfully were dissected, and the heads/thoraces separated from the abdomens immediately, as explained above. The presence of sporozoite was investigated using a TaqMan genotyping protocol, established by Bass and colleagues [33]. Real-time PCR MX 3005 (Agilent, Santa Clara, CA, USA) was used for the amplification. A total of 1 μL of gDNA, extracted from each female head/thorax, was used for PCR, with an initial denaturation at 95 °C for 10 min, followed by 40 cycles each of 15 s at 95 °C and 1 min at 60 °C. Primers described by Bass (PlasF_GCTTAGTTACGATTAATAGGAGTAGCTTG and PlasR_GAAAATCTAAGAATTTCACCTCTGACA) were used, together with two probes labelled with fluorophores, FAM (Falcip+_TCTGAATACGAATGTC) to detect *Plasmodium falciparum*, and HEX (OVM+_CTGAATACAAATGCC) to detect the combination of *P. ovale*, *P. vivax* and *P. malariae*. Positive controls (known FAM+ and OVM+) were used, in addition to a negative control, in which 1 μL of ddH_2_O was added. To validate findings of the TaqMan assay, a nested PCR of Snounou et al. [34] was carried out, using all the samples that tested positive with TaqMan. The sporozoite rate was calculated as the percentage of females positive, relative to the total number of the females examined [30]. 

### 2.4. WHO Insecticides Susceptibility Tests

The bioassays were performed following the protocol of WHO [35], with various insecticides from four major public health classes. These include (i) the type I pyrethroid: permethrin (0.75%); (ii) the type II pyrethroid: deltamethrin (0.05%); (iii) the organochloride: DDT (4%); (iv) the carbamate: bendiocarb (0.1%); and (v) the organophosphate: fenitrothion (1%). All insecticide impregnated papers (reference: WHO/VBC/81.806) were sourced from the WHO/Vector Control Research Unit (VCRU) of University of Sains Malaysia (Penang, Malaysia). Four replicates of 24–26 F_1_ females (3–4 d old) per tube were used for each insecticide. The mosquitoes were transferred to tubes lined with insecticide papers and exposed for 1 h, after which they were transferred back to the holding tubes, supplied with 10% sugar, and mortality recorded at 24 h. For each bioassay, one replicate of 20–25 females unexposed to any insecticides were used as a control. The fully susceptible *An. funestus* (FANG colony) [36] was tested, alongside the field populations, for the pyrethroid papers to ascertain the potency of the papers. The mosquitoes were deemed susceptible to an insecticide where mortality was >98%, suspected to be moderately resistant where mortality is between 90–98%, and resistant where mortality was <90% [35]. Figures were prepared using GraphPad Prism 7.02 (GraphPad Inc., La Jolla, CA, USA).

### 2.5. Estimation of Resistance Intensity with Time-Course Bioassay

To establish the strength of pyrethroid resistance with time, additional bioassays were performed, with 0.75% permethrin, at varying times of exposure. Four replicates of 20–25 F_1_ females were exposed in time-course spanning 60, 120, 180, 240 and 300 min, to establish the time required to kill 50% of them (LT_50_). Protocol was as described above in conventional bioassays, except for variation in time. Resistance intensity was established by comparing the LT_50_ from the Gajerar Giwa *An. funestus* populations to the LT_50_ previously established for the fully susceptible *Anopheles coluzzii* lab colony [5]. This type of inter-species comparison of using *An. gambiae* (Kisumu colony) LT_50_s had been done before in the absence of LT_50_ data from FANG colony [37]. 

### 2.6. Determination of LLIN Efficacy with Cone Bioassays 

To investigate the efficacy of commonly distributed long-lasting insecticidal nets (LLINs), cone bioassays were conducted following the WHO protocol [36], using 3–4 d old F_1_ females. Five replicates of 9–12 mosquitoes were placed in a plastic cone attached to a fresh insecticide-containing bed net and tested. The LLINs include: the Olyset^®^Net (containing 2% permethrin), Olyset^®^Plus (containing 2% permethrin combined with 1% of the synergist, piperonyl butoxide, PBO), PermaNet^®^2.0 (containing 1.4–1.8 g/kg ± 25% deltamethrin), PermaNet^®^3.0 side panel (containing 2.1–2.8 g/kg ± 25% deltamethrin), and PermaNet^®^3.0 roof (containing 4.0 g/kg ± 25% deltamethrin, combined with 25 g/kg ± 25% of PBO). For each experiment, five different pieces cut from an LLIN brand were used for the five technical replicates. Mosquitoes were exposed for 3 min and immediately transferred to paper cups. They were supplied with 10% sucrose and mortalities recorded after 24 h. For the control, five replicates of ten mosquitoes were exposed to untreated net.

### 2.7. Synergist Bioassay with Piperonyl Butoxide (PBO) and Diethyl Maleate

To investigate the potential role of P450 monooxygenases in pyrethroid resistance, a synergist bioassay was carried out using 4% PBO [an inhibitor of CYP450s [38]] against permethrin. The role of glutathione S-transferases (GSTs) in DDT resistance was also investigated by pre-exposure to 8% diethyl maleate (DEM). The insecticides and the synergists PBO were sourced from the WHO/Vector Control Research Unit (VCRU) of the University of Sains Malaysia (Penang, Malaysia). Four replicates of 22–26 F_1_ females (3–4 d old) were pre-exposed to PBO or DEM for 1 h, and then transferred to tubes containing permethrin [35] or DDT, respectively. Mosquitoes were treated as in the WHO bioassays described above, and mortalities scored after 24 h. Two replicates of 25 females each were exposed to PBO only, as a control.

### 2.8. Genotyping of L119F Glutathione S-Transferase (GSTe2) Mutation Associated with DDT/Permethrin Resistance 

To detect the L119F *GSTe2* mutation previously linked to DDT/pyrethroid metabolic resistance [20], TaqMan genotyping was conducted using 44 F_0_ females collected from the field. This was carried out using a real-time PCR thermocycler (Agilent Mx3005) following an established protocol [20]. The primers L119F_*GSTe2*F (5′-AACAATTTTTCATTTCTTATTCTCATTTAC-3′) and L119F_*GSTe2*R (5′- CGACTCGATCTTCGGGAATGTC-3′) were utilised with the following probes: reporter L119 (VIC_AGGAGCGTATTCTTTTCTA) to detect the susceptibility allele, and reporter 119F (FAM_AGGAGCGTATTTTTTTCTA) for the resistance allele. The assay was performed in a 10 µL final volume comprise of 5 µL of 1x Sensimix (Bioline, London, UK), 800 nM each of primer, 200 nM of each probe, and 1 µL of gDNA. Thermocycling conditions were initial denaturation of 10 min at 95 °C, followed by 40 cycles each of 92 °C for 10 s, and 60 °C for 45 s. Genotypes were scored from scatter plots of results produced by the Mx3005 v4.10 software (Agilent, Santa Clara, CA, USA). Three positive samples of known genotypes [20]—(i) homozygote resistant 1014F/1014F; (ii) heterozygote L1014/1014F; and (iii) susceptible L1014/L1014—were used as positive controls. For the negative control, 1 µL of ddH_2_O was incorporated into the control well. To assess the correlation between the presence of the 119F mutation and the resistance phenotype, 12 each of DDT-alive and -dead females were genotyped using the above protocol. In addition, these 12 alive and 12 dead females were also screened, using the recently established allele-specific PCR [39].

### 2.9. Polymorphism Analysis of Acetylcholinesterase-1 (ace-1) for G119S and N485I Target-Site Mutations 

To detect the 119S *ace-1* mutation linked to bendiocarb/organophosphate resistance [23] a fragment of the gene spanning exons 4 to 5 was amplified from 12 bendiocarb-alive and -12 dead females. The following primers spanning exon 4, through intron 4_5 and exon 5: AFUNace1_ex4-5F: 5′-ACGCTAACGATAATGATCCGCT-3′ and AFUNace1_ex4-5R:5′AGTAGCTTCTTCGCGTGATA CA-3′ were utilised. A 12.5 μL premix comprise of 2x AccuStar II PCR SuperMix (QuantaBio, Beverly, Massachusetts, USA), containing optimised concentrations of MgCl_2_ and dNTPs, 0.2 μmol/L each of the forward and reverse primer was prepared. A total of 1 μL gDNA from individual female mosquitoes was added, followed by 10.5 μL of ddH_2_O (final volume = 25 μL). Amplification was carried out using the following condition: initial denaturation of 1 cycle at 94 °C for 3 min; followed by 35 cycles each of 94 °C for 30 s (denaturation); 60 °C for 30 s (annealing); extension at 72 °C for 1 min; and final elongation of one cycle at 72 °C for 5 min. PCR products were cleaned with QIAquick^®^ PCR Purification Kit (QIAGEN, Hilden, Germany) and sequenced on both strands, using the above primers. 

For the N485I mutation the same samples as above were used. A forward primer AfunExon6-7ace_F: 5’-AACAATGTATACATGTACCT-3’ was utilised together with a previously described reverse primer AfunExon4-7ace R: 5’-TGACACTAGCAGCACAACCA-3’ [24] to amplify a fragment spanning exons 6–7. Thermocycling condition was as described above. 

Polymorphisms were detected through manual examination of sequence traces using BioEdit version 7.2.3.0 (http://www.mbio.ncsu.edu/BioEdit/bioedit.html) [40], and analyses of genetic parameters of polymorphism were done using the DnaSP 5.10 [41]. Different sequences were compared by constructing a maximum likelihood phylogenetic tree using MEGA 6.0 [42]. All DNA sequences from the alive and dead females are provided in File S1.

### 2.10. Polymorphism Analysis of Voltage-Gated Sodium Channel Gene Spanning the 1014 kdr Locus

A fragment of voltage-gated sodium channel spanning intron 19 and the exon 20 containing the 1014F/S mutations associated with knockdown resistance (*kdr*) in *An. gambiae* [21,22] was amplified and sequenced. This was done with DNA extracted from 30 F_0_ females that laid eggs successfully. PCR was carried using primers: AfunkdrExon19-20F: GTTCAATGAAGCCCCTCAAA and Afunkdr Exon19-20R: CCGAAATTTGACAAAAGCAAA, as described in previous works [43]. The PCR products were purified using the Qiaquick Purification Kit (QIAGEN, Hilden, Germany), sequenced and aligned using BioEdit. Polymorphisms analysis and maximum likelihood phylogenetic tree construction were as described above. All DNA sequences from the alive and dead females are provided in File S1.

### 2.11. Data Analysis

The results of bioassays were interpreted as continuous variables, with normal distributions and percentage mortalities ± standard error of mean (SEM) calculated, based on the WHO protocol [35]. Results of mortalities from synergist-pyrethroid exposure were compared with values obtained from exposure to pyrethroid alone using a two-tailed Chi-Square test of independence, with the level of significance pegged as *P* < 0.05, as implemented in GraphPad Prism 7.02 (GraphPad Inc., La Jolla, CA, USA). For genotyping of 119F *GSTe2* mutation allele frequency was calculated using the formula f(R) = (2 × RR + RS)/2*N* for individuals carrying the mutations, and f(S) = 1 − f(R) for the susceptible individuals; where RR = total number of homozygote resistant; RS = total number heterozygote resistant; *N*, total number of individuals investigated. Genotype frequency was calculated as relative frequencies of the homozygote resistant and heterozygote resistant individuals. The correlation between the genotypes and resistance phenotypes was estimated by estimating the odds ratio using the epiR package [R version 3.5.0 (https://cran.r-project.org/bin/windows/base/)] with statistical significance established based on the Fisher’s exact probability test (*P* < 0.05). For the estimation of LT_50_, a probit analysis was carried out using glm with a MASS package of R [44].

### 2.12. Ethical Approval

Clearance for indoor collection previously obtained on 12/01/2017 from Operational Research Advisory Committee, Ministry of Health (with reference number MOH/off/797/TI/402), Kano state was used. A meeting was held with the village head and household members at Gajerar Giwa; the scope of the study and its benefits to malaria control was explained. A written and signed informed consent was obtained from individuals who agreed to indoor collection in their houses. All communication was carried out in the local language (Hausa). 

## 3. Results

### 3.1. Composition of the Mosquito Species Collected Indoor

*Anopheles funestus* is the only mosquito species caught, a total of 721 in 4 d. Out of these, 590 were caught in the first 3 d (52 ♂, 538 ♀ [487 blood fed and 51 unfed]). A total of 312 ♀ laid eggs and 288 egg batches hatched successfully. A total of 131 mosquitoes were caught on the 4th day: 12 ♂ and 119 ♀ (112 of them blood fed and 7 unfed). 

### 3.2. Entomological and Parasitological Parameters of Transmission

#### 3.2.1. Indoor Resting Density, Human Blood Index and Biting Rate

The indoor resting density of the *An. funestus* was estimated as 14 from the 112 blood females collected in eight houses. Analysis of the blood source of these 112 females by PCR revealed that they all fed on human blood, leading to a human blood index of 100%. The human biting rate was estimated as ~5.3 bites per person per night (CI: 4.91–5.69). 

#### 3.2.2. Estimation of Sporozoite Infection Rate 

A total of 36 out of 66 heads/thoraces were positive for *P. falciparum* (F_+_ = 36), corresponding to an unusually high sporozoite rate of 54.55% ± 13.63. Validation of the TaqMan results using nested PCR [34] confirmed the *P. falciparum* infection in the 36 samples. 

### 3.3. Insecticide Resistance Profile of An. funestus Population

**Figure 2:**
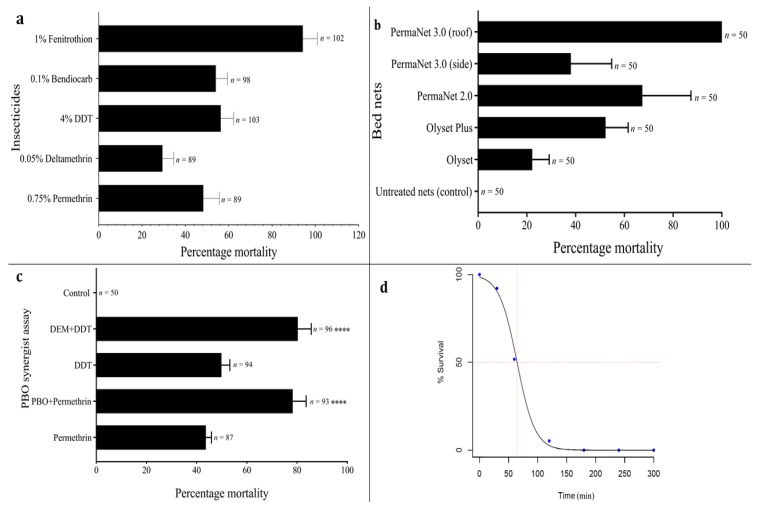
Resistance profiles of F_1_ *An. funestus* females. (**a**). Results of WHO susceptibility bioassays with various insecticides. Results are the average of percentage mortalities from four replicates each ± standard error of mean (SEM); (**b**). Results of the cone bioassays with PermaNet^®^3.0 (side and roof), PermaNet^®^2.0, Olyset^®^Plus and Olyset^®^Net. Results are the average of percentage mortalities ± SEM of five replicates; (**c**). Effect of pre-exposure of synergist piperonylbutoxide (PBO) against permethrin and diethyl maleate (DEM) against DDT (dichlorodiphenyltrichloroethane). Results are the average of percentage mortalities from four replicates each ± SEM. **** = statistically significant at *P* < 0.0001, in a two-tailed Chi-square test between results from synergised bioassay and conventional bioassays; (**d**). Time-course bioassay for LT_50_ estimation/test for strength of permethrin resistance.

The *An. funestus* population demonstrated high knockdown resistance, with ~5% of them knocked down at 1 h exposure with deltamethrin and DDT (Figure S1), and ~10% knocked down after 1 h exposure with permethrin. High resistance was observed towards the pyrethroids, with a higher mortality of 48.30% (95% CI: 41–55) for permethrin, compared with 29.44% (95% CI: 24–34) for deltamethrin (Figure 2a). A 100% mortality rate was obtained in the FANG susceptible colony. Significant resistance was also observed towards DDT and bendiocarb, with mortalities of 56.34% (95% CI: 51–62) and 54.05% (95% CI: 49–59), respectively. Moderate resistance was observed from exposure to fenitrothion with mortality of 94.22% (95% CI: 88–101). 

### 3.4. Pyrethroid Resistance Intensity 

The LT_50_ for permethrin was estimated as 64.76 min [95% CI: 59.17–70.35, Fiducial] (Figure 2d) after assessing mortality from 30 to 300 min exposure. To calculate the resistance intensity, the LT_50_ from Gajerar Giwa *An. funestus* was compared to LT_50_ (3.320 min), previously established for the fully susceptible *An. coluzzii* Ngoussou lab colony [5]. The resistance ratio was estimated as 19.51. 

### 3.5. Bed Net Efficacy Using Cone Bioassay

A low efficacy was observed with the pyrethroid-based Olyset^®^Net (mortality = 22.22, 95% CI: 14.37–30.07) and the Perma^®^Net2.0 (mortality = 38.05%, CI: 19.12–56.98) (Figure 2b). The side panels of PermaNet^®^3.0 induced higher mortality of 67.41% (CI: 44.87–89.95). This is more than the mortality observed with the PBO-containing Olyset^®^Plus (mortality = 52.31%, CI: 41.85–62.78). A 100% mortality rate was seen from exposure to the roof of PermaNet^®^3.0 (containing PBO). No mortality was obtained from the control populations, which were exposed to untreated nets.

### 3.6. Investigating the Role of Metabolic Resistance Using Synergist Bioassays 

Pre-exposure to PBO and DEM recovered some susceptibility to both permethrin and DDT, respectively (Figure 2c). A significant increase in mortality was observed when comparing results of a repeat conventional bioassay with permethrin [mortality = 43.9% (95% CI: 40.93–46.90)] to the results from the PBO-synergised bioassay [mortality = 78.73% (95% CI: 69.53–87.94), χ^2^ = 22.33, df = 1, *P* < 0.0001]. Pre-exposure to DEM recovered DDT susceptibility as well, with repeat exposure to DDT producing mortality of 49.82% (95% CI: 45.54–54.9), compared to a synergised experiment with a mortality of 81.44% (95% CI: 74.36–88.52, χ^2^ = 19.12, df = 1, *P* < 0.0001). In both cases, the doubling of mortalities from synergist pre-exposure suggests the possible role of cytochrome P450s and GSTs, respectively, in the observed permethrin and DDT resistance. No mortality was obtained from the control individuals. 

### 3.7. Investigating the Role of L119F-GSTe2 Mutation in DDT Resistance 

**Figure 3:**
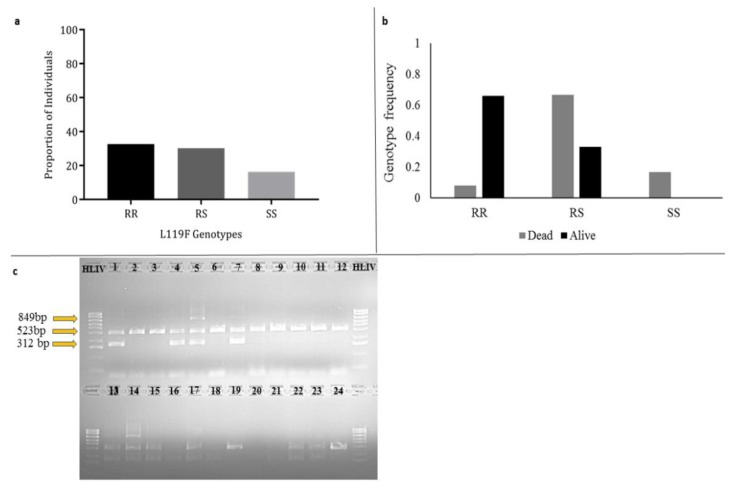
Genotyping of L119F GSTe2 mutation. (**a**): distribution of the 119F mutation among the 44 randomly selected F_0_ females; (**b**): the 119F mutation genotype for 12 each of DDT-alive and -dead F_1_ females, RR = homozygote resistant, RS = heterozygote resistant, and SS = susceptible; (**c**): agarose-gel showing the L119F genotype distribution from allele-specific PCR (AS-PCR), top panel: 12 DDT-alive F_1_ females (2, 3, 6, and 8–12 are RR, 1, 4, 5 and 7 are RS), bottom panel: 12 DDT-dead F_1_ females (13, 14, 15, 17, 19, 21–24 are RS, 16 and 18 are SS, and 20 failed).

Initial genotyping of 43 F_0_ females revealed a high frequency of the L119F-GSTe2 mutation with 30.2% (13 individuals) heterozygotes (RS, L119/F119), 32.6% (14 individuals) homozygote resistant (RR, 119F/119F), and 16.3% (7 individuals) homozygote susceptible (SS, L119/L119) (Figure 3a). 

The 119F allele frequency f(R) was calculated as ~0.84. 12 each of DDT-alive and -dead F_1_ females were used for allele-specific PCR to detect the L119F GSTe2 mutation. 8 of the DDT-alive (8/12, 66.66%) were homozygote resistant (RR) for the mutation (Figure 3b,c), and 4 females (4/12, 33.33%) were heterozygote resistant. From the dead females, one sample failed (Figure 3c), only one female was homozygote resistant (1/11, 9.09%), 8 were heterozygotes (8/11, 72.72%), and 2 were susceptible (2/11, 18.18%). A difference was observed in the distribution of the 119F mutation [Odds Ratio of 16.00 (95% CI: 6.67–38.3, χ^2^ = 3.70, *p* = 0.05)] when comparing the frequency of the homozygote resistant allele (RR) with susceptible allele (SS) in the alive and dead females. A comparison of all resistant (RR + RS) with susceptible (SS) individuals from alive and dead females revealed no genotype-phenotype association [OR = 0.49 (CI: 0.19–3.15, χ^2^ = 1.2, *p* = 0.27)]. No association was observed when comparing the heterozygote individuals (RS) from both alive and dead to the susceptible (SS) [OR = 2.44 (CI: 0.9–13.53, χ^2^ = 0.49, *p* = 0.48)]. 

### 3.8. Polymorphism Analysis of the ace-1 Fragment Encompassing the G119S and N485I Resistance Mutations

An 806 bp fragment of *ace-1* gene encompassing exons 4–5 was successfully sequenced. Analysis of this fragment reveals absence of the G119S mutation in 12 bendiocarb-alive and 12 -dead females (wild type codon GGA found). The *ace-1* fragment was found to be highly polymorphic, with up to 17 haplotypes from 24 sequences, 23 polymorphic sites, and a high haplotype diversity of 0.95 (Figure S2a, Table S1). Only two non-synonymous mutations were seen in a single sequence from one dead female (a G -> T substitution in nucleotide number 800 leading to replacement of glutamic acid with lysine, and a G -> A nucleotide substitution in nucleotide number 803 leading to replacement of alanine to threonine). Also, the sequences did not cluster according to their phenotype in a maximum likelihood phylogenetic tree (Figure S2b). All sequences are provided in File S1.

The 485I *ace-1* mutation was also absent in the 12 bendiocarb-alive and 12 -dead females (AAC codon for asparagine was found in all sequences). The 609 bp fragments spanning exons 6–9 were analysed from all sequences and found to be highly polymorphic, with 23 haplotypes, 23 polymorphic sites, and a very high haplotype diversity of 0.98 (Figure S3a, Table S2). No non-synonymous polymorphism was discovered in all sequences. These sequences also did not cluster according to phenotypes in the maximum likelihood phylogenetic tree (Figure S3b). These sequences are provided in File S1.

### 3.9. Polymorphism Analysis of the Voltage-Gated Sodium Channel Encompassing the L1014F/S Knockdown Resistance Mutation

Analysis of the 907 bp fragment spanning the 1014 codon established the absence of the 1014F/S *kdr* mutation in exon 20 from 30 field-collected females. The 30 sequences share 25 haplotypes (Figure S4a, Table S3), with 43 polymorphic sites, and a very high haplotype diversity of 0.97. All sequences have the TTA codon for leucine in the 1014 position. No other mutations were found in the coding region. Sequences are highly heterogenous, as evidenced in the maximum likelihood phylogenetic tree. The sequences have been provided in File S1.

## 4. Discussion

The successful control and pre-elimination of malaria in Nigeria will benefit greatly from the entomological surveillance of the major malaria vectors in the heterogenous regions of the country (with different eco-climatic attributes), their role in transmission, and their resistance profiles. This is especially important for the major malaria vector *An. funestus*, which has been neglected in the country. To facilitate the control of malaria in the Sahel of northern Nigeria, the role of a population of *An. funestus* in transmission of *Plasmodium* and its resistance profile was characterised. The possible molecular mechanisms driving the resistance were also investigated. 

### 4.1. Evidence of Unusually High Transmission Capability by Gajerar Giwa An. funestus 

The finding of *An. funestus* as the only malaria vector in the dry season at Gajerar Giwa is in agreement with previous observations that the density of this species peak in the dry season [13,18], allowing it to extend the period of malaria transmission when the densities of *An. gambiae* s.l. have declined [14]. The high anthropophilic and endophilic behaviour of this species [14,15] probably explain the high indoor resting densities observed in this study, compared to previous observations [12], and the 100% preference for human blood, with the latter probably augmented by lack of insecticide treated bed nets in Gajerar Giwa. Indeed, several studies [45], including one from southern Nigeria [17], have documented a very high human blood index in *An. funestus*. Initial studies carried out in Nigeria have described a sporozoite rate of less than 5% for this species. These include the comprehensive work carried out at Garki [12], and the work of Leonard and Bruce-Chwatt in 1951 [13], as well as the 2005 report by Awolola on this species from southern Nigeria [17]. The transmission capability of this species has skyrocketed since then, with a *P. falciparum* sporozoite rate of ~55% found in this study, which is comparable to the 57% obtained by Magellan and colleagues on the same species from northern Cameroon, although at the oocyst stage [39]. However, a study has described lower sporozoite rates of ~8% for this species but from southern Nigeria [18]. This high infection rate possibly explains the high burden of malaria seen in Nigeria, especially as seen in the Sahel region, in non-rainy season, when malaria transmission is supposed to be at its lowest. 

### 4.2. Evidence of Multiple Resistance in the Gajerar Giwa An. funestus

The multiple insecticide resistance seen in the *An. funestus* in this study is in line with reported data from several studies across Africa [20,46,47,48], though with the intensity of the resistance higher in southern Africa. However, the level of pyrethroid resistance observed in this study was higher than reported in the same species from southern Nigeria by Djouaka et al. [18], especially for deltamethrin, where mortalities in the population from southern Nigeria was on average three times lower compared to the Gajerar Giwa population. In contrast, the extremely high DDT resistance reported in southern Nigeria [18] was not seen in the Gajerar Giwa population, though the DDT resistance is higher compared to the values reported for the same species in Sahel of northern Senegal [48]. The high bendiocarb resistance seen in the Gajerar Giwa population (54% mortality) is lower than carbamate resistance in southern African populations (30–42% mortality) [37,46], where carbamate resistance is established to be very high. However, the bendiocarb resistance in the Gajerar Giwa is higher than what was reported for the southern Nigerian population of *An. funestus* (84% mortality) [18], or the population from Senegal (94% mortality) [48], and even in northern Cameroon (89% mortality) [49]. The Gajerar Giwa *An. funestus* population exhibited a high LT_50_ for permethrin, reflecting high resistance intensity, though on average less than half of what was documented for the southern African populations [37,46]. 

### 4.3. A Low-Efficacy of Long-Lasting Insecticidal Nets Is a Serious Challenge to Malaria Control 

The high pyrethroid resistance was also evident in the low efficacy of insecticide-treated bed nets, most especially the Olyset^®^Net and the PermaNet^®^3.0 (the side panel). Except for PermaNet^®^3.0, profoundly lower efficacies were seen with all the nets, in line with observations in same species from southern Africa [46], and a recent report from northern Cameroon [50]. The recovery of susceptibilities from exposure to the roof of PermaNet^®^3.0 and the PBO-synergist bioassays suggest the preeminent role of CYP450s in the pyrethroid resistance in this population. This agrees with several studies carried out across Africa [46,51,52]. Low efficacy seen with Olyset^®^Plus net have been described in several studies with both *An. funestus* and *An. coluzzii* [50,51,52,53].

### 4.4. Insecticide Resistance Mechanism Is Driven by Metabolic Resistance Mechanisms in Gajerar Giwa An. funestus Population 

The recovery of susceptibilities from pre-exposure to PBO and DEM suggests the preeminent role of CYP450s and GSTs in pyrethroid and DDT resistance, respectively. Indeed, several studies have used these synergists to establish the potential role of the above metabolic enzymes in resistance in *An. funestus* [51,52,54]. The role of metabolic resistance is strengthened by (i) the finding of high frequency of the 119F–*GSTe2* mutation in the Gajerar Giwa population and its possible link to DDT resistance, as documented in several studies [20,47,51,54]; (ii) the absence of the G119S and N485I mutations in the acetylcholinesterase gene; and (iii) the absence of the 1014F/S *kdr* mutation in the voltage-gated sodium channel from the Gajerar Giwa population, in line with the observations across Africa [37,52,55]. The high diversity seen in the fragment spanning the exon 20 of the VGSC suggests lack of reduced diversity due to absence of selection. 

The high bendiocarb resistance prompted sequencing of the *ace-1* fragment for the G119S and N485I mutations previously associated with carbamate/organophosphate resistance. The absence of these mutations in the highly bendiocarb-resistant Gajerar Giwa populations, and the observed relative fenitrothion susceptibility, suggests no cross resistance, and points to metabolic mechanism responsible for the bendiocarb resistance. Several studies have reported absence of the G119S mutation in various populations of *An. funestus* across Africa [24,43,52]. The high diversity seen in the *ace-1* fragment suggests a lack of selection in the gene. However, with only 12 females (low sample size) each of alive and dead used, the presence of this mutation at a low frequency cannot be ruled out. 

The absence of the N485I mutation in Gajerar Giwa populations confirmed previous observations of the mutation present only in southern African populations [24,46]. However, with 12 females each of the alive and dead used, the presence of these mutations or others at a low frequency cannot be ruled out. 

## 5. Conclusions

This study discovered a disproportionately high *Plasmodium* infection in a major malaria vector *An. funestus* from Nigeria, which will pose a serious threat to malaria control, and highlights the extent of the efforts needed to reach the pre-elimination stage in this region of Nigeria. Pyrethroid and DDT resistance was seen at high levels which could compromise control tools using the LLINs and indoor residual spraying (IRS). The high bendiocarb resistance observed in the field populations of this vector may affect the future control measures in northern Nigeria, using the carbamate-based IRS. However, the PBO-containing combination bed net PermaNet^®^3.0 was found to be still effective in killing this species. The discovery of a high susceptibility to fenitrothion suggests that organophosphates could also be alternatives for IRS. Finally, all evidences pointed to the preeminent role of metabolic mechanisms in the multiple resistance in this populations of *An. funestus*. This should be considered by Nigeria’s National Malaria Elimination Program when deploying control tools/interventions into this area. 

## Supplementary Materials

The following are available online at https://www.mdpi.com/2073-4425/11/4/454/s1, Figure S1: Knockdown profiles of *An. funestus females* exposed to pyrethroids and DDT. Each bar is a percentage knockdown of values from four different replicates from bioassays at different time points, Figure S2: Analysis of the *ace-1* fragment encompassing the 119 codon. (**a**). pattern of genetic variability of sequences from 12 bendiocarb-alive and -12 dead females; (**b**). a maximum likelihood phylogenetic tree of the sequences., Figure S3: Analysis of the *ace-1* fragment encompassing the 485 codon. (**a**). pattern of genetic variability of sequences from 12 bendiocarb-alive and -12 dead females; (**b**). a maximum likelihood phylogenetic tree of the sequences., Figure S4: Analysis of the *VGSC* fragment encompassing the 1014 *kdr* mutation codon. (**a**). pattern of genetic variability of 30 sequences from randomly selected F_0_ females; (**b**). a maximum likelihood phylogenetic tree of the sequences, Table S1: Summary statistics for polymorphism of a fragment of *acetylcholinesterase*-1 encompassing the 119 codon for alive and dead mosquitoes, Table S2: Summary statistics for polymorphism of a fragment of *acetylcholinesterase*-1 encompassing the 485 codon for alive and dead mosquitoes, Table S3: Summary statistics for polymorphism of a fragment of voltage-gated sodium channel encompassing the 1014 codon for the field F_0_ females, File S1: Analyzed sequences (gDNA partial fragments) from encompassing the G119S and N485I of *acetylcholinesterase*-1 gene and sequences from the partial fragment of the voltage-gated sodium channel spanning the 1014th codon.
