# Supplementary material for: Exploring the Mechanisms of Multiple Insecticide Resistance in a Highly Plasmodium-Infected Malaria Vector Anopheles funestus Sensu Stricto from Sahel of Northern Nigeria
Source: Genes (Basel). 2020 Apr 22;11(4):454. doi: 10.3390/genes11040454 (PMC7230678; doi:10.3390/genes11040454)
Supplement: Supplementary file 1 [file genes-11-00454-s001.zip › supplementary/File S1.docx]

**Exploring mechanisms of multiple insecticide resistance in a highly *Plasmodium*-infected malaria vector *Anopheles funestus* sensu stricto from Sahel of northern Nigeria**

**Sulaiman S. Ibrahim, Muhammad M. Mukhtar, Helen Irving, Jacob M. Riveron, Amen N. Fadel, William Tchapga, Jack Hearn, Abdullahi Muhammad, Faruk Sarkinfada and Charles S. Wondji**

Nigeria *An. funestus acetylcholinesterase-*1 G119 partial gDNA sequences

>NIG_AL1_G119 [organism=Anopheles funestus] [Nigeria] ace-1 gDNA, partial CDS

GCTAACGATAATGATCCGCTGGTAGTTAACACCGACAAGGGTCGTATT

CGCGGCATCACCGTGGAAGCACCGAGCGGCAAAAAGGTAGATGTGTGGCT

TGGCATTCCCTACGCACAGCCACCGGTGGGGCCGCTACGATTCCGTCATC

CCCGACCGGCCGAAAAGTGGAATGGTGTGCTTAATGCCACCACACCGCCC

AACAGCTGCGTACAGATCGTGGACACCGTGTTCGGTGACTTTCCGGGTGC

GACCATGTGGAACCCGAACACACCACTGTCGGAGGACTGTCTGTACATTA

ATGTGGTAGCACCACGACCGCGACCGAAGAATGCTGCCGTTATGCTGTGG

ATCTTTGGCGGTGGATTTTACTCCGGTACCGCTACGCTCGACGTGTACGA

TCACCGTGCGCTCGCATCGGAGGAGAATGTGATCGTCGTCTCGCTACAGT

ACCGGGTAGCTAGTTTGGGTTTCCTGTTTCTCGGCACACCGGAAGCGCCG

GGCAATGCGGGACTGTTCGATCAGAACCTTGCACTACGGTAGGATTTCTG

TGGAGGTGTGCTATCAGCGATTAGTGTAACTAATGATCTGCTCTTTCGCA

TCAACAAATCCGCAGCTGGGTACGGGACAACATTCACAGGTTCGGTGGTG

ATCCGTCGCGTGTAACACTGTTCGGGGAGAGTGCCGGTGCGGTATCCGTT

TCGCTTCATCTGCTGTCCGCACTTTCGCGTGATCTGTTCCAACGTGCCAT

ACTTCAGAGCGGTTCACCGACGGCACCATGGGCGCTTGTATCACGCGAAG

AAGCTACT

>NIG_AL2_G119 [organism=Anopheles funestus] [Nigeria] ace-1 gDNA, partial CDS

GCTAACGATAATGATCCGCTGGTAGTTAACACCGACAAGGGTCGTATT

CGCGGCATCACCGTGGAAGCACCGAGCGGCAAAAAGGTAGATGTGTGGCT

TGGCATTCCCTACGCACAGCCACCGGTTGGGCCGCTACGATTCCGTCATC

CCCGACCGGCCGAAAAGTGGAATGGTGTGCTTAATGCCACCACACCGCCC

AACAGCTGCGTACAGATCGTGGACACCGTGTTCGGTGACTTTCCGGGTGC

GACCATGTGGAACCCGAACACACCACTGTCGGAGGACTGTCTGTACATTA

ATGTGGTAGCACCACGACCGCGACCGAAGAATGCTGCCGTTATGCTGTGG

ATCTTTGGCGGTGGATTTTACTCCGGTACCGCTACGCTCGACGTGTACGA

TCACCGTGCGCTCGCATCGGAAGAGAATGTGATCGTCGTCTCGCTACAGT

ACCGGGTAGCTAGTTTGGGTTTCCTGTTTCTCGGCACACCGGAAGCGCCG

GGCAATGCGGGACTGTTCGATCAGAACCTTGCACTACGGTAGGATTTCTG

TGGAGGTGTGCTATCAGCGATAAGTGTAACTAATGATCTGCTCTTTCGCA

TCAACAAATCCGCAGCTGGGTACGGGACAACATTCACAGGTTCGGTGGTG

ATCCGTCGCGTGTAACACTGTTCGGGGAGAGTGCCGGTGCGGTATCCGTT

TCGCTTCATCTGCTGTCCGCACTTTCGCGTGATCTGTTCCAACGTGCCAT

ACTTCAGAGCGGTTCACCGACGGCACCATGGGCGCTTGTATCACGCGAAG

AAGCTACT

>NIG_AL3_G119 [organism=Anopheles funestus] [Nigeria] ace-1 gDNA, partial CDS

GCTAACGATAATGATCCGCTGGTAGTTAACACCGACAAGGGTCGTATT

CGCGGCATCACCGTGGAAGCACCGAGCGGCAAAAAGGTAGATGTGTGGCT

TGGCATTCCCTACGCACAGCCACCGGTTGGGCCGCTACGATTCCGTCATC

CCCGACCGGCCGAAAAGTGGAATGGTGTGCTTAATGCCACCACACCGCCC

AACAGCTGCGTACAGATCGTGGACACCGTGTTCGGTGACTTTCCGGGTGC

GACCATGTGGAACCCGAACACACCACTGTCGGAGGACTGTCTGTACATTA

ATGTGGTAGCACCACGACCGCGACCGAAGAATGCTGCCGTTATGCTGTGG

ATCTTTGGCGGTGGATTTTACTCCGGTACCGCTACGCTCGACGTGTACGA

TCACCGTGCGCTCGCATCGGAAGAGAATGTGATCGTCGTCTCGCTACAGT

ACCGGGTAGCTAGTTTGGGTTTCCTGTTTCTCGGCACACCGGAAGCGCCG

GGCAATGCGGGACTGTTCGATCAGAACCTTGCACTACGGTAGGATTTCTG

TGGAGGTGTGCTATCAGCGATTAGTGTAACTAATGATCTGCTCTTTTGCA

TCAACAAATCCGCAGCTGGGTACGGGACAACATTCACAGGTTCGGTGGTG

ATCCGTCGCGTGTAACACTGTTCGGGGAGAGTGCCGGTGCGGTATCCGTT

TCGCTTCATCTGCTGTCCGCACTTTCGCGTGATCTGTTCCAGCGTGCCAT

ACTTCAGAGCGGTTCACCGACGGCACCATGGGCGCTTGTATCACGCGAAG

AAGCTACT

>NIG_AL4_G119 [organism=Anopheles funestus] [Nigeria] ace-1 gDNA, partial CDS

GCTAACGATAATGATCCGCTGGTAGTTAACACCGACAAGGGTCGTATT

CGCGGCATCACCGTGGAAGCACCGAGCGGCAAAAAGGTAGATGTGTGGCT

TGGCATTCCCTACGCACAGCCACCGGTTGGGCCGCTACGATTCCGTCATC

CCCGACCGGCCGAAAAGTGGAATGGTGTGCTTAATGCCACCACACCGCCC

AACAGCTGCGTACAGATCGTGGACACCGTGTTCGGTGACTTTCCGGGTGC

GACCATGTGGAACCCGAACACACCACTGTCGGAGGACTGTCTGTACATTA

ATGTGGTAGCACCACGACCGCGACCGAAGAATGCTGCCGTTATGCTGTGG

ATCTTTGGCGGTGGATTTTACTCCGGTACCGCTACGCTCGACGTGTACGA

TCACCGTGCGCTCGCATCGGAAGAGAATGTGATCGTCGTCTCGCTACAGT

ACCGGGTAGCTAGTTTGGGTTTCCTGTTTCTCGGCACACCGGAAGCGCCG

GGCAATGCGGGACTGTTCGATCAGAACCTTGCACTACGGTAGGATTTCTG

TGGAGGTGTGCTATCAGCGATTAGTGTAACTAATGATCTGCTCTTTTGCA

TCAACAAATCCGCAGCTGGGTACGGGACAACATTCACAGGTTCGGTGGTG

ATCCGTCGCGTGTAACACTGTTCGGGGAGAGTGCCGGTGCGGTATCCGTT

TCGCTTCATCTGCTGTCCGCACTTTCGCGTGATCTGTTCCAGCGTGCCAT

ACTTCAGAGCGGTTCACCGACGGCACCATGGGCGCTTGTATCACGCGAAG

AAGCTACT

>NIG_AL5_G119 [organism=Anopheles funestus] [Nigeria] ace-1 gDNA, partial CDS

GCTAACGATAATGATCCGCTGGTAGTTAACACCGACAAGGGTCGTATT

CGCGGCATCACCGTGGAAGCACCGAGCGGCAAAAAGGTAGATGTGTGGCT

TGGCATTCCCTACGCACAGCCACCGGTTGGGCCGCTACGATTCCGTCATC

CCCGACCGGCCGAAAAGTGGAATGGTGTGCTTAATGCCACCACACCGCCC

AACAGCTGCGTACAGATCGTGGACACCGTGTTCGGTGACTTTCCGGGTGC

GACCATGTGGAACCCGAACACACCACTGTCGGAGGACTGTCTGTACATTA

ATGTGGTAGCACCACGACCGCGACCGAAGAATGCTGCCGTTATGCTGTGG

ATCTTTGGCGGTGGATTTTACTCCGGTACCGCTACGCTCGATGTGTACGA

TCACCGTGCGCTCGCATCGGAGGAGAACGTGATCGTCGTCTCGCTACAGT

ACCGGGTAGCTAGTTTGGGTTTCCTGTTTCTCGGCACACCGGAAGCGCCG

GGCAATGCGGGACTGTTCGATCAGAACCTTGCACTACGGTAGGATTTCTG

TGGAGGTGTGCTATCAGCGATTAGTGTAACTAATGATCTGCTCTTTCGCA

TCAACAAATCCGCAGCTGGGTACGGGACAACATTCACAGGTTCGGTGGTG

ATCCGTCGCGTGTAACACTGTTCGGGGAGAGTGCCGGTGCGGTATCCGTT

TCGCTTCATCTGCTGTCCGCACTTTCGCGTGATCTGTTCCAGCGTGCCAT

ACTTCAGAGCGGTTCACCGACGGCACCATGGGCGCTTGTATCACGCGAAG

AAGCTACT

>NIG_AL6_G119 [organism=Anopheles funestus] [Nigeria] ace-1 gDNA, partial CDS

GCTAACGATAATGATCCGCTGGTAGTTAACACCGACAAGGGTCGTATT

CGCGGCATCACCGTGGAAGCACCGAGCGGCAAAAAGGTAGATGTGTGGCT

TGGCATTCCCTACGCACAGCCACCGGTTGGGCCGCTACGATTCCGTCATC

CCCGACCGGCCGAAAAGTGGAATGGTGTGCTTAATGCCACCACACCGCCC

AACAGCTGCGTACAGATCGTGGACACCGTGTTCGGTGACTTTCCGGGTGC

GACCATGTGGAACCCGAACACACCACTGTCGGAGGACTGTCTGTACATTA

ATGTGGTAGCACCACGACCGCGACCGAAGAATGCTGCCGTTATGCTGTGG

ATCTTTGGCGGTGGATTTTACTCCGGTACCGCTACGCTCGACGTGTACGA

TCACCGTGCGCTCGCATCGGAGGAGAATGTGATCGTCGTCTCGCTACAGT

ACCGGGTAGCTAGTTTGGGTTTCCTGTTTCTCGGCACACCGGAAGCGCCG

GGCAATGCGGGACTGTTCGATCAGAACCTTGCACTACGGTAGGATTTCTG

TGGAGGTGTGCTATCAGCGATAAGTGTAACTAATGATCTGCTCTTTCGCA

TCAACAAATCCGCAGCTGGGTACGGGACAACATTCACAGGTTCGGTGGTG

ATCCGTCGCGTGTAACACTGTTCGGGGAGAGTGCCGGTGCGGTATCCGTT

TCGCTTCATCTGCTGTCCGCACTTTCGCGTGATCTGTTCCAACGTGCCAT

ACTTCAGAGCGGTTCACCGACGGCACCATGGGCGCTTGTATCACGCGAAG

AAGCTACT

>NIG_AL7_G119 [organism=Anopheles funestus] [Nigeria] ace-1 gDNA, partial CDS

GCTAACGATAATGATCCGCTGGTAGTTAACACCGACAAGGGTCGTATT

CGCGGCATCACCGTGGAAGCACCGAGCGGCAAAAAGGTAGATGTGTGGCT

TGGCATTCCCTACGCACAGCCACCGGTTGGGCCGCTACGATTCCGTCATC

CCCGACCGGCCGAAAAGTGGAATGGTGTGCTTAATGCCACCACACCGCCC

AACAGCTGCGTACAGATCGTGGACACCGTGTTCGGTGACTTTCCGGGTGC

GACCATGTGGAACCCGAACACACCACTGTCGGAGGACTGTCTGTACATTA

ATGTGGTAGCACCACGACCGCGACCGAAGAATGCTGCCGTTATGCTGTGG

ATCTTTGGCGGTGGATTTTACTCCGGTACCGCTACGCTCGATGTGTACGA

TCACCGTGCGCTCGCATCGGAGGAGAACGTGATCGTCGTCTCGCTACAGT

ACCGGGTAGCTAGTTTGGGTTTCCTGTTTCTCGGCACACCGGAAGCGCCG

GGCAATGCGGGACTGTTCGATCAGAACCTTGCACTACGGTAGGATTTCTG

TGGAGGTGTGCTATCAGCGATTAGTGTAACTAATGATCTGCTCTTTCGCA

TCAACAAATCCGCAGCTGGGTACGGGACAACATTCACAGGTTCGGTGGTG

ATCCGTCGCGTGTAACACTGTTCGGGGAGAGTGCCGGTGCGGTATCCGTT

TCGCTTCATCTGCTGTCCGCACTTTCGCGTGATCTGTTCCAGCGTGCCAT

ACTTCAGAGCGGTTCACCGACGGCACCATGGGCGCTTGTATCACGCGAAG

AAGCTACT

>NIG_AL8_G119 [organism=Anopheles funestus] [Nigeria] ace-1 gDNA, partial CDS

GCTAACGATAATGATCCGCTGGTAGTTAACACCGACAAGGGTCGTATT

CGCGGCATCACCGTGGAAGCACCGAGCGGCAAAAAGGTAGATGTGTGGCT

TGGCATTCCCTACGCACAGCCACCGGTTGGGCCGCTACGATTCCGTCATC

CCCGACCGGCCGAAAAGTGGAATGGTGTGCTTAATGCCACCACACCGCCC

AACAGCTGCGTACAGATCGTGGACACCGTGTTCGGTGACTTTCCGGGTGC

GACCATGTGGAACCCGAACACACCACTGTCGGAGGACTGTCTGTACATTA

ATGTGGTAGCACCACGACCGCGACCGAAGAATGCTGCCGTTATGCTGTGG

ATCTTTGGCGGTGGATTTTACTCCGGTACCGCTACGCTCGACGTGTACGA

TCACCGTGCGCTCGCATCGGAGGAGAATGTGATCGTCGTCTCGCTACAGT

ACCGGGTAGCTAGTTTGGGTTTCCTGTTTCTCGGCACACCGGAAGCGCCG

GGCAATGCGGGACTGTTCGATCAGAACCTTGCACTACGGTAGGATTTCTG

TGGAGGTGTGCTATCAGCGATAAGTGTAACTAATGATCTGCTCTTTCGCA

TCAACAAATCCGCAGCTGGGTACGGGACAACATTCACAGGTTCGGTGGTG

ATCCGTCGCGTGTAACACTGTTCGGGGAGAGTGCCGGTGCGGTATCCGTT

TCGCTTCATCTGCTGTCCGCACTTTCGCGTGATCTGTTCCAACGTGCCAT

ACTTCAGAGCGGTTCACCGACGGCACCATGGGCGCTTGTATCACGCGAAG

AAGCTACT

>NIG_AL9_G119 [organism=Anopheles funestus] [Nigeria] ace-1 gDNA, partial CDS

GCTAACGATAATGATCCGCTGGTAGTTAACACCGACAAGGGTCGTATT

CGCGGCATCACCGTGGAAGCACCGAGCGGCAAAAAGGTAGATGTGTGGCT

TGGCATTCCCTACGCACAGCCACCGGTTGGGCCGCTACGATTCCGTCATC

CCCGACCGGCCGAAAAGTGGAATGGTGTGCTTAATGCCACCACACCGCCC

AACAGCTGCGTACAGATCGTGGACACCGTGTTCGGTGACTTTCCGGGTGC

GACCATGTGGAACCCGAACACACCACTGTCGGAGGACTGTCTGTACATTA

ATGTGGTAGCACCACGACCGCGACCGAAGAATGCTGCCGTTATGCTGTGG

ATCTTTGGCGGTGGATTTTACTCCGGTACCGCTACGCTCGACGTGTACGA

TCACCGTGCGCTCGCATCGGAGGAGAACGTGATCGTCGTCTCGCTACAGT

ACCGGGTAGCTAGTTTGGGTTTCCTGTTTCTCGGCACACCGGAAGCGCCG

GGCAATGCGGGACTGTTCGATCAGAACCTTGCACTACGGTAGGATTTCTG

TGGAGGTGTGCTATCAGCGATTAGTGTAACTAATGATCTGCTCTTTCGCA

TCAACAAATCCGCAGCTGGGTACGGGACAACATTCACAGGTTCGGTGGTG

ATCCGTCGCGTGTAACACTGTTCGGGGAGAGTGCCGGTGCGGTATCCGTT

TCGCTTCATCTGCTGTCCGCACTTTCGCGTGATCTGTTCCAACGTGCCAT

ACTTCAGAGCGGTTCACCGACGGCACCATGGGCGCTTGTATCACGCGAAG

AAGCTACT

>NIG_AL10_G119 [organism=Anopheles funestus] [Nigeria] ace-1 gDNA, partial CDS

GCTAACGATAATGATCCGCTGGTAGTTAACACCGACAAGGGTCGTATT

CGCGGCATCACCGTGGAAGCACCGAGCGGCAAAAAGGTAGATGTGTGGCT

TGGCATTCCCTACGCACAGCCACCGGTTGGGCCGCTACGATTCCGTCATC

CCCGACCGGCCGAAAAGTGGAATGGTGTGCTTAATGCCACCACACCGCCC

AACAGCTGCGTACAGATCGTGGACACCGTGTTCGGTGACTTTCCGGGTGC

GACCATGTGGAACCCGAACACACCACTGTCGGAGGACTGTCTGTACATTA

ATGTGGTAGCACCACGACCCCGACCGAAGAATGCTGCCGTTATGCTGTGG

ATCTTTGGCGGTGGATTTTACTCCGGTACCGCTACGCTCGACGTGTACGA

TCACCGTGCGCTCGCATCGGAGGAGAATGTGATCGTCGTCTCGCTACAGT

ACCGGGTAGCTAGTTTGGGTTTCCTGTTTCTCGGCACACCGGAAGCGCCG

GGCAATGCGGGACTGTTCGATCAGAACCTTGCACTACGGTAGGATTTCTG

TGGAGGTGTGCTATCAGCGATAAGTGTAACTAATGATCTGCTCTTTCGCA

TCAACAAATCCGCAGCTGGGTACGGGACAACATTCACAGGTTCGGTGGTG

ATCCGTCGCGTGTAACACTGTTCGGGGAGAGTGCCGGTGCGGTATCCGTT

TCGCTTCATCTGCTGTCCGCACTTTCGCGTGATCTGTTCCAACGTGCCAT

ACTTCAGAGCGGTTCACCGACGGCACCATGGGCGCTTGTATCACGCGAAG

AAGCTACT

>NIG_AL11_G119 [organism=Anopheles funestus] [Nigeria] ace-1 gDNA, partial CDS

GCTAACGATAATGATCCGCTGGTAGTTAACACCGACAAGGGTCGTATT

CGCGGCATCACCGTGGAAGCACCGAGCGGCAAAAAGGTAGATGTGTGGCT

TGGCATTCCCTACGCACAGCCACCGGTTGGGCCGCTACGATTCCGTCATC

CCCGACCGGCCGAAAAGTGGAATGGTGTGCTTAATGCCACCACACCGCCC

AACAGCTGCGTACAGATCGTGGACACCGTGTTCGGTGACTTTCCGGGTGC

GACCATGTGGAACCCGAACACACCACTGTCGGAGGACTGTCTGTACATTA

ATGTGGTAGCACCACGACCGCGACCGAAGAATGCTGCCGTTATGCTGTGG

ATCTTTGGCGGTGGATTTTACTCCGGTACCGCTACGCTCGACGTGTACGA

TCACCGTGCGCTCGCATCGGAAGAGAATGTGATCGTCGTCTCGCTACAGT

ACCGGGTAGCTAGTTTGGGTTTCCTGTTTCTCGGCACACCGGAAGCGCCG

GGCAATGCGGGACTGTTCGATCAGAACCTTGCACTACGGTAGGATTTCTG

TGGAGGTGTGCTATCAGCGATTAGTGTAACTAATGATCTGCTCTTTCGCA

TCAACAAATCCGCAGCTGGGTACGGGACAACATTCACAGGTTCGGTGGTG

ATCCGTCGCGTGTAACACTGTTCGGGGAGAGTGCCGGTGCGGTATCCGTT

TCGCTTCATCTGCTGTCCGCACTTTCGCGTGATCTGTTCCAACGTGCCAT

ACTTCAGAGCGGTTCACCGACGGCACCATGGGCGCTTGTATCACGCGAAG

AAGCTACT

>NIG_AL12_G119 [organism=Anopheles funestus] [Nigeria] ace-1 gDNA, partial CDS

GCTAACGATAATGATCCGCTGGTAGTTAACACCGACAAGGGTCGTATT

CGCGGCATCACCGTGGAAGCACCGAGCGGCAAAAAGGTAGATGTGTGGCT

TGGCATTCCCTACGCACAGCCACCGGTTGGGCCGCTACGATTCCGTCATC

CCCGACCGGCCGAAAAGTGGAATGGTGTGCTTAATGCCACCACACCGCCC

AACAGCTGCGTACAGATCGTGGACACCGTGTTCGGTGACTTTCCGGGTGC

GACCATGTGGAACCCGAACACACCACTGTCGGAGGACTGTCTGTACATTA

ATGTGGTAGCACCACGACCGCGACCGAAGAATGCTGCCGTTATGCTGTGG

ATCTTTGGCGGTGGATTTTACTCCGGTACCGCTACGCTCGACGTGTACGA

TCACCGTGCGCTCGCATCGGAGGAGAACGTGATCGTCGTCTCGCTACAGT

ACCGGGTAGCTAGTTTGGGTTTCCTGTTTCTCGGCACACCGGAAGCGCCG

GGCAATGCGGGACTGTTCGATCAGAACCTTGCACTACGGTAGGATTTCTG

TGGAGGTGTGCTATCAGCGATTAGTGTAACTAATGATCTGCTCTTTCGCA

TCAACAAATCCGCAGCTGGGTACGGGACAACATTCACAGGTTCGGTGGTG

ATCCGTCGCGTGTAACACTGTTCGGGGAGAGTGCCGGTGCGGTATCCGTT

TCGCTTCATCTGCTGTCCGCACTTTCGCGTGATCTGTTCCAACGTGCCAT

ACTTCAGAGCGGTTCACCGACGGCACCATGGGCGCTTGTATCACGCGAAG

AAGCTACT

>NIG_DE1_G119 [organism=Anopheles funestus] [Nigeria] ace-1 gDNA, partial CDS

GCTAACGATAATGATCCGCTGGTAGTTAACACCGACAAGGGTCGTATT

CGCGGCATCACCGTGGAAGCACCGAGCGGCAAAAAGGTGGATGTGTGGCT

TGGCATTCCCTACGCACAGCCACCGGTTGGGCCGCTACGATTCCGTCATC

CCCGACCGGCCGAAAAGTGGAATGGTGTGCTTAATGCCACCACACCGCCC

AACAGCTGCGTACAGATCGTGGACACCGTGTTCGGTGACTTTCCGGGTGC

GACCATGTGGAACCCGAACACACCACTGTCGGAGGACTGTCTGTACATTA

ATGTGGTAGCACCACGACCGCGACCGAAGAATGCTGCCGTTATGCTGTGG

ATCTTTGGCGGTGGATTTTACTCCGGTACCGCTACGCTCGACGTGTACGA

TCACCGTGCGCTCGCATCGGAGGAGAACGTGATCGTCGTCTCGCTACAGT

ACCGGGTAGCTAGTTTGGGTTTCCTGTTTCTCGGCACACCGGAAGCGCCG

GGCAATGCGGGACTGTTCGATCAGAACCTTGCACTACGGTAGGATTTCTG

TGGAGGTGTGCTATCAGCGATTAGTGTAACTAATGATCTGCTCTTTCGCA

TCAACAAATCCGCAGCTGGGTACGGGACAACATTCACAGGTTCGGTGGTG

ATCCGTCGCGAGTAACACTGTTCGGGGAGAGTGCCGGTGCGGTATCCGTT

TCGCTTCATCTGCTGTCCGCACTTTCGCGTGATCTGTTCCAACGTGCCAT

ACTTCAGAGCGGTTCACCGACGGCACCATGGGCGCTTGTATCACGCGAAG

AAGCTACT

>NIG_DE2_G119 [organism=Anopheles funestus] [Nigeria] ace-1 gDNA, partial CDS

GCTAACGATAATGATCCGCTGGTAGTCAACACCGACAAGGGTCGTATT

CGCGGCATCACCGTGGAAGCACCGAGCGGCAAAAAGGTAGATGTGTGGCT

TGGCATTCCCTACGCACAGCCACCGGTTGGGCCGCTACGATTCCGTCATC

CCCGACCGGCCGAAAAGTGGAATGGTGTGCTTAATGCCACCACACCGCCC

AACAGCTGCGTACAGATCGTGGACACCGTGTTCGGTGACTTTCCGGGTGC

GACCATGTGGAACCCGAACACACCACTGTCGGAGGACTGTCTGTACATTA

ATGTGGTAGCACCACGACCGCGACCGAAGAATGCTGCCGTTATGCTGTGG

ATCTTTGGCGGTGGATTTTACTCCGGTACCGCTACGCTCGACGTGTACGA

TCACCGTGCGCTCGCATCGGAGGAGAACGTGATCGTCGTCTCGCTACAGT

ACCGGGTAGCTAGTTTGGGTTTCCTGTTTCTCGGCACACCGGAAGCGCCG

GGCAATGCGGGACTGTTCGATCAGAACCTTGCACTACGGTAGGATTTCTG

TGGAGGTGTGCTATCAGCGATTAGTGTAACTAATGATCTGCTCTTTCGCA

TCAACAAATCCGCAGCTGGGTACGGGACAACATTCACAGGTTCGGTGGTG

ATCCGTCGCGTGTAACACTGTTCGGGGAGAGTGCCGGTGCGGTATCCGTT

TCGCTTCATCTGCTGTCCGCACTTTCGCGTGATCTGTTCCAACGTGCCAT

ACTTCAGAGCGGTTCACCGACGGCACCATGGGCGCTTGTATCACGCGAAG

AAGCTACT

>NIG_DE3_G119 [organism=Anopheles funestus] [Nigeria] ace-1 gDNA, partial CDS

GCTAACGATAATGATCCGCTGGTAGTTAACACCGACAAGGGTCGTATT

CGCGGCATCACCGTGGAAGCACCGAGCGGCAAAAAGGTAGATGTGTGGCT

TGGCATTCCCTACGCACAGCCACCGGTTGGGCCGCTACGATTCCGTCATC

CCCGACCGGCCGAAAAGTGGAATGGTGTGCTTAATGCCACCACACCGCCC

AACAGCTGCGTACAGATCGTGGACACCGTGTTCGGTGACTTTCCGGGTGC

GACCATGTGGAACCCGAACACACCACTGTCGGAGGACTGTCTGTACATTA

ATGTGGTAGCACCACGACCGCGACCGAAGAATGCTGCCGTTATGCTGTGG

ATCTTTGGCGGTGGATTTTACTCCGGTACCGCTACGCTCGACGTGTACGA

TCACCGTGCGCTCGCATCGGAAGAGAACGTGATCGTCGTCTCGCTACAGT

ACCGGGTAGCTAGTTTGGGTTTCCTGTTTCTCGGCACACCGGAAGCGCCG

GGCAATGCGGGACTGTTCGATCAGAACCTTGCACTACGGTAGGATTTCTG

TGGAGGTGTGCTATCAGCGATTAGTGTAACTAATGATCTGCTCTTTCGCA

TCAACAAATCCGCAGCTGGGTACGGGACAACATTCACAGGTTCGGTGGTG

ATCCGTCGCGTGTAACACTGTTCGGGGAGAGTGCCGGTGCGGTATCCGTT

TCGCTTCATCTGCTGTCCGCACTTTCGCGTGATCTGTTCCAACGTGCCAT

ACTTCAGAGCGGTTCACCGACGGCACCATGGGCGCTTGTATCACGCGAAG

AAGCTACT

>NIG_DE4_G119 [organism=Anopheles funestus] [Nigeria] ace-1 gDNA, partial CDS

GCTAACGATAATGATCCGCTGGTAGTTAACACCGACAAGGGTCGTATT

CGCGGCATCACCGTGGAAGCACCGAGCGGCAAAAAGGTAGATGTGTGGCT

TGGCATTCCCTACGCACAGCCACCGGTTGGGCCGCTACGATTCCGTCATC

CCCGACCGGCCGAAAAGTGGAATGGTGTGCTTAATGCCACCACACCGCCC

AACAGCTGCGTACAGATCGTGGACACCGTGTTCGGTGACTTTCCGGGTGC

GACCATGTGGAACCCGAACACACCACTGTCGGAGGACTGTCTGTACATTA

ATGTGGTAGCACCACGACCGCGACCGAAGAATGCTGCCGTTATGCTGTGG

ATCTTTGGCGGTGGATTTTACTCCGGTACCGCTACGCTCGACGTGTACGA

TCACCGTGCGCTCGCATCGGAAGAGAACGTGATCGTCGTCTCGCTACAGT

ACCGGGTAGCTAGTTTGGGTTTCCTGTTTCTCGGCACACCGGAAGCGCCG

GGCAATGCGGGACTGTTCGATCAGAACCTTGCACTACGGTAGGATTTCTG

TGGAGGTGTGCTATCAGCGATTAGTGTAACTAATGATCTGCTCTTTCGCA

TCAACAAATCCGCAGCTGGGTACGGGACAACATTCACAGGTTCGGTGGTG

ATCCGTCGCGTGTAACACTGTTCGGGGAGAGTGCCGGTGCGGTATCCGTT

TCGCTTCATCTGCTGTCCGCACTTTCGCGTGATCTGTTCCAACGTGCCAT

ACTTCAGAGCGGTTCACCGACGGCACCATGGGCGCTTGTATCACGCGAAG

AAGCTACT

>NIG_DE5_G119 [organism=Anopheles funestus] [Nigeria] ace-1 gDNA, partial CDS

GCTAACGATAATGATCCGCTGGTAGTTAACACCGACAAGGGTCGTATT

CGCGGCATCACCGTGGAAGCACCGAGCGGCAAAAAGGTAGATGTGTGGCT

TGGCATTCCCTACGCACAGCCACCGGTTGGGCCGCTACGATTCCGTCATC

CCCGACCGGCCGAAAAGTGGAATGGTGTGCTTAATGCCACCACACCGCCC

AACAGCTGCGTACAGATCGTGGACACCGTGTTCGGTGACTTTCCGGGTGC

GACCATGTGGAACCCGAACACACCACTGTCGGAGGACTGTCTGTACATTA

ATGTGGTAGCACCACGACCGCGACCGAAGAATGCTGCCGTTATGCTGTGG

ATCTTTGGCGGTGGATTTTACTCCGGTACCGCTACGCTCGACGTGTACGA

TCACCGTGCGCTCGCATCGGAAGAGAACGTGATCGTCGTCTCGCTACAGT

ACCGGGTAGCTAGTTTGGGTTTCCTGTTTCTCGGCACACCGGAAGCGCCG

GGCAATGCGGGACTGTTCGATCAGAACCTTGCACTACGGTAGGATTTCTG

TGGAGGTGTGCTATCAGCGATTAGTGTAACTAATGATCTGCTCTTTCGCA

TCAACAAATCCGCAGCTGGGTACGGGACAACATTCACAGGTTCGGTGGTG

ATCCGTCGCGTGTAACACTGTTCGGGGAGAGTGCCGGTGCGGTATCCGTT

TCGCTTCATCTGCTGTCCGCACTTTCGCGTGATCTGTTCCAACGTGCCAT

ACTTCAGAGCGGTTCACCGACGGCACCATGGGCGCTTGTATCACGCGAAG

AAGCTACT

>NIG_DE6_G119 [organism=Anopheles funestus] [Nigeria] ace-1 gDNA, partial CDS

GCTACGGATAATGATCCGCTGGTAGTTAACACCGACAAGGGTCGTATT

CGCGGCATCACCGTGGAAGCACCGAGCGGCAAAAAGGTAGATGTGTGGCT

TGGCATTCCCTACGCACAGCCACCGGTTGGGCCGCTACGATTCCGTCATC

CCCGACCGGCCGAAAAGTGGAATGGTGTGCTTAATGCCACCACACCGCCC

AACAGCTGCGTACAGATCGTGGACACCGTGTTCGGTGACTTTCCGGGTGC

GACCATGTGGAACCCGAACACACCACTGTCGGAGGACTGTCTGTACATTA

ATGTGGTAGCACCACGACCGCGACCGAAGAATGCTGCCGTTATGCTGTGG

ATCTTTGGCGGTGGATTTTACTCCGGTACCGCTACGCTCGACGTGTACGA

TCACCGTGCGCTCGCATCGGAGGAGAATGTGATCGTCGTCTCGCTACAGT

ACCGGGTAGCTAGTTTGGGTTTCCTGTTTCTCGGCACACCGGAAGCGCCG

GGCAATGCGGGACTGTTCGATCAGAACCTTGCACTACGGTAGGATTTCTG

TGGAGGTGTGCTATCAGCGATTAGTGTAACTAATGATCTGCTCTTTCGCA

TCAACAAATCCGCAGCTGGGTACGGGACAACATTCACAGGTTCGGTGGTG

ATCCGTCGCGTGTAACACTGTTCGGGGAGAGTGCCGGTGCGGTATCCGTT

TCGCTTCATCTGCTGTCCGCACTTTCGCGTGATCTGTTCCAACGTGCCAT

ACTTCAGAGCGGTTCACCGACGGCACCATGGGCGCTTGTATCACGCGAAG

AAGCTACT

>NIG_DE7_G119 [organism=Anopheles funestus] [Nigeria] ace-1 gDNA, partial CDS

GCTAACGGATATGATCCGCTGGTAGTTAACACCGACAAGGGTCGTATT

CGCGGCATCACCGTGGAAGCACCGAGCGGCAAAAAGGTAGATGTGTGGCT

TGGCATTCCCTACGCACAGCCACCGGTTGGGCCGCTACGATTCCGTCATC

CCCGACCGGCCGAAAAGTGGAATGGTGTGCTTAATGCCACCACACCGCCC

AACAGCTGCGTACAGATCGTGGACACCGTGTTCGGTGACTTTCCGGGTGC

GACCATGTGGAACCCGAACACACCACTGTCGGAGGACTGTCTGTACATTA

ATGTGGTAGCACCACGACCGCGACCGAAGAATGCTGCCGTTATGCTGTGG

ATCTTTGGCGGTGGATTTTACTCCGGTACCGCTACGCTCGACGTGTACGA

TCACCGTGCGCTCGCATCGGAAGAGAACGTGATCGTCGTCTCGCTACAGT

ACCGGGTAGCTAGTTTGGGTTTCCTGTTTCTCGGCACACCGGAAGCGCCG

GGCAATGCGGGACTGTTCGATCAGAACCTTGCACTACGGTAGGATTTCTG

TGGAGGTGTGCTATCAGCGATTAGTGTAACTAATGATCTGCTCTTTCGCA

TCAACAAATCCGCAGCTGGGTACGGGACAACATTCACAGGTTCGGTGGTG

ATCCGTCGCGTGTAACACTGTTCGGGGAGAGTGCCGGTGCGGTATCCGTT

TCGCTTCATCTGCTGTCCGCACTTTCGCGTGATCTGTTCCAACGTGCCAT

ACTTCAGAGCGGTTCACCGACGGCACCATGGGCGCTTGTATCACGCGAAG

AAGCTACT

>NIG_DE8_G119 [organism=Anopheles funestus] [Nigeria] ace-1 gDNA, partial CDS

GCTAACGATAATGATCCGCTGGTAGTTAACACCGACAAGGGTCGTATT

CGCGGCATCACCGTGGAAGCACCGAGCGGCAAAAAGGTAGATGTGTGGCT

TGGCATTCCCTACGCACAGCCACCGGTTGGGCCGCTACGATTCCGTCATC

CCCGACCGGCCGAAAAGTGGAATGGTGTGCTTAATGCCACCACACCGCCC

AACAGCTGCGTACAGATCGTGGACACCGTGTTCGGTGACTTTCCGGGTGC

GACCATGTGGAACCCGAACACACCACTGTCGGAGGACTGTCTGTACATTA

ATGTGGTAGCACCACGACCGCGACCGAAGAATGCTGCCGTTATGCTGTGG

ATCTTTGGCGGTGGATTTTACTCCGGTACCGCTACGCTCGACGTGTACGA

TCACCGTGCGCTCGCATCGGAGGAGAACGTGATCGTCGTCTCGCTACAGT

ACCGGGTAGCTAGTTTGGGTTTCCTGTTTCTCGGCACACCGGAAGCGCCG

GGCAATGCGGGACTGTTCGATCAGAACCTTGCACTACGGTAGGATTTCTG

TGGAGGTGTGCTATCAGCGATTAGTGTAACTAATGATCTGCTCTTTCGCA

TCAACAAATCCGCAGCTGGGTACGGGACAACATTCACAGGTTCGGTGGTG

ATCCGTCGCGTGTAACACTGTTCGGGGAGAGTGCCGGTGCGGTATCCGTT

TCGCTTCATCTGCTGTCCGCACTTTCGCGTGATCTGTTCCAGCGTGCCAT

ACTTCAGAGCGGTTCACCGACGGCACCATGGGCGCTTGTATCACGCGAAG

AAGCTACT

>NIG_DE9_G119 [organism=Anopheles funestus] [Nigeria] ace-1 gDNA, partial CDS

GCAACCGGATATGATCCGCTGGTAGTTAACACCGACAAGGGTCGTATT

CGCGGCATCACCGTGGAAGCACCGAGCGGCAAAAAGGTAGATGTGTGGCT

TGGCATTCCCTACGCACAGCCACCGGTTGGGCCGCTACGATTCCGTCATC

CCCGACCGGCCGAAAAGTGGAATGGTGTGCTTAATGCCACCACACCGCCC

AACAGCTGCGTACAGATCGTGGACACCGTGTTCGGTGACTTTCCGGGTGC

GACCATGTGGAACCCGAACACACCACTGTCGGAGGACTGTCTGTACATTA

ATGTGGTAGCACCACGACCGCGACCGAAGAATGCTGCCGTTATGCTGTGG

ATCTTTGGCGGTGGATTTTACTCCGGTACCGCTACGCTCGACGTGTACGA

TCACCGTGCGCTCGCATCGGAGGAGAACGTGATCGTCGTCTCGCTACAGT

ACCGGGTAGCTAGTTTGGGTTTCCTGTTTCTCGGCACACCGGAAGCGCCG

GGCAATGCGGGACTGTTCGATCAGAACCTTGCACTACGGTAGGATTTCTG

TGGAGGTGTGCTATCAGCGATTAGTGTAACTAATGATCTGCTCTTTCGCA

TCAACAAATCCGCAGCTGGGTACGGGACAACATTCACAGGTTCGGTGGTG

ATCCGTCGCGTGTAACACTGTTCGGGGAGAGTGCCGGTGCGGTATCCGTT

TCGCTTCATCTGCTGTCCGCACTTTCGCGTGATCTGTTCCAACGTGCCAT

ACTTCAGAGCGGTTCACCGACTGCACCATGGGCGCTTGTATCACGCGAAA

AAACTACT

>NIG_DE10_G119 [organism=Anopheles funestus] [Nigeria] ace-1 gDNA, partial CDS

GCTAACGATAATGATCCGCTGGTAGTTAACACCGACAAGGGTCGTATT

CGCGGCATCACCGTGGAAGCACCGAGCGGCAAAAAGGTAGATGTGTGGCT

TGGCATTCCCTACGCACAGCCACCGGTTGGGCCGCTACGATTCCGTCATC

CCCGACCGGCCGAAAAGTGGAATGGTGTGCTTAATGCCACCACACCGCCC

AACAGCTGCGTACAGATCGTGGACACCGTGTTCGGTGACTTTCCGGGTGC

GACCATGTGGAACCCGAACACACCACTGTCGGAGGACTGTCTGTACATTA

ATGTGGTAGCACCACGACCCCGACCGAAGAATGCTGCCGTTATGCTGTGG

ATCTTTGGCGGTGGATTTTACTCCGGTACCGCTACGCTCGACGTGTACGA

TCACCGTGCGCTCGCATCGGAAGAGAATGTGATCGTGGTCTCGCTACAGT

ACCGGGTAGCTAGTTTGGGTTTCCTGTTTCTCGGCACACCGGAAGCGCCG

GGCAATGCGGGACTGTTCGATCAGAACCTTGCACTACGGTAGGATTTCTG

TGAAGGTGCGCTATCAGCGATTAGTGTAACTAATGATCTGCTCTTTCGCA

TCAACAAATCCGCAGCTGGGTACGGGACAACATTCACAGGTTCGGTGGTG

ATCCGTCGCGAGTAACACTGTTCGGGGAGAGTGCCGGTGCGGTATCCGTT

TCGCTTCATCTGCTGTCCGCACTTTCGCGTGATCTGTTCCAACGTGCCAT

ACTTCAGAGCGGTTCACCGACGGCACCATGGGCGCTTGTATCACGCGAAG

AAGCTACT

>NIG_DE11_G119 [organism=Anopheles funestus] [Nigeria] ace-1 gDNA, partial CDS

GCTAACGATAATGATCCGCTGGTAGTTAACACCGACAAGGGTCGTATT

CGCGGCATCACCGTGGAAGCACCGAGCGGCAAAAAGGTAGATGTGTGGCT

TGGCATTCCCTACGCACAGCCACCGGTTGGGCCGCTACGATTCCGTCATC

CCCGACCGGCCGAAAAGTGGAATGGTGTGCTTAATGCCACCACACCGCCC

AACAGCTGCGTACAGATCGTGGACACCGTGTTCGGTGACTTTCCGGGTGC

GACCATGTGGAACCCGAACACACCACTGTCGGAGGACTGTCTGTACATTA

ATGTGGTAGCACCACGACCCCGACCGAAGAATGCTGCCGTTATGCTGTGG

ATCTTTGGCGGTGGATTTTACTCCGGTACCGCTACGCTCGACGTGTACGA

TCACCGTGCGCTCGCATCGGAAGAGAACGTGATCGTCGTCTCGCTACAGT

ACCGGGTAGCTAGTTTGGGTTTCCTGTTTCTCGGCACACCGGAAGCGCCG

GGCAATGCGGGACTGTTCGATCAGAACCTTGCACTACGGTAGGATTTCTG

TGGAGGTGTGCTATCAGCGATTAGTGTAACTAATGATCTGCTCTTTCGCA

TCAACAAATCCGCAGCTGGGTACGGGACAACATTCACAGGTTCGGTGGTG

ATCCGTCGCGTGTAACACTGTTCGGGGAGAGTGCCGGTGCGGTATCCGTT

TCGCTTCATCTGCTGTCCGCACTTTCGCGTGATCTGTTCCAGCGTGCCAT

ACTTCAGAGCGGTTCACCGACGGCACCATGGGCGCTTGTATCACGCGAAG

AAGCTACT

>NIG_DE12_G119 [organism=Anopheles funestus] [Nigeria] ace-1 gDNA, partial CDS

GCTAACGATAATGATCCGCTGGTAGTTAACACCGACAAGGGTCGTATT

CGCGGCATCACCGTGGAAGCACCGAGCGGCAAAAAGGTAGATGTGTGGCT

TGGCATTCCCTACGCACAGCCACCGGTTGGGCCGCTACGATTCCGTCATC

CCCGACCGGCCGAAAAGTGGAATGGTGTGCTTAATGCCACCACACCGCCC

AACAGCTGCGTACAGATCGTGGACACCGTGTTCGGTGACTTTCCGGGTGC

GACCATGTGGAACCCGAACACACCACTGTCGGAGGACTGTCTGTACATTA

ATGTGGTAGCACCACGACCCCGACCGAAGAATGCTGCCGTTATGCTGTGG

ATCTTTGGCGGTGGATTTTACTCCGGTACCGCTACGCTCGACGTGTACGA

TCACCGTGCGCTCGCATCGGAAGAGAACGTGATCGTCGTCTCGCTACAGT

ACCGGGTAGCTAGTTTGGGTTTCCTGTTTCTCGGCACACCGGAAGCGCCG

GGCAATGCGGGACTGTTCGATCAGAACCTTGCACTACGGTAGGATTTCTG

TGGAGGTGTGCTATCAGCGATTAGTGTAACTAATGATCTGCTCTTTCGCA

TCAACAAATCCGCAGCTGGGTACGGGACAACATTCACAGGTTCGGTGGTG

ATCCGTCGCGTGTAACACTGTTCGGGGAGAGTGCCGGTGCGGTATCCGTT

TCGCTTCATCTGCTGTCCGCACTTTCGCGTGATCTGTTCCAGCGTGCCAT

ACTTCAGAGCGGTTCACCGACGGCACCATGGGCGCTTGTATCACGCGAAG

AAGCTACT

Nigeria *An. funestus acetylcholinesterase-*1 N485 partial gDNA sequences

>NIG_AL1_N485 [organism=Anopheles funestus] [Nigeria] ace-1 gDNA, partial CDS

AACAATGTATACATGTACCTGTACACGCACCGAAGCAAAGGCAACCCATG

GCCACGCTGGACGGGCGTCATGCACGGTGATGAGATTAACTATGTGTTCG

GGGAACCGCTCAATCCCAGCCTCGGCTACACCGAGGACGAGAAAGACTTT

AGCCGAAAGATCATGCGATACTGGTCAAACTTTGCCAAGACGGGGTAGGA

TTCTTAGCAATATAACGCATTAACTATTTCTAACGATTCCATTTCCCATC

ATTTTGCCCTTCCAGCAATCCAAACCCCAACACGGCTAGTAGCGAATTTC

CCGAATGGCCGAAACATACGGCCCACGGACGGCACTATCTGGAGCTGGGC

CTCAACACGTCCTTCGTCGGACGGGGTCCCCGGTTGAGGCAGTGTGCCTT

CTGGAAGAAATATCTTCCCCAGCTAGTTGCAGCTACCTGTTAGTATCTAC

GTCACGTAAGATGTAACGCTTTCGAAACAAATAAACGTTTCTAAACCAAT

GCCTTCGTGTTCATTCGCAGCTAACATTGGAGGTGAACCACTACCAAGTG

CACCGTGCGAAAGCAGCGCATTTTTTTACCGACCTGATCTGGTTGTGCTG

CTAGTGTCA

>NIG_AL2_N485 [organism=Anopheles funestus] [Nigeria] ace-1 gDNA, partial CDS

AACAATGTATACATGTACCTGTACACGCACCGAAGCAAAGGCAACCCATG

GCCACGCTGGACGGGCGTTATGCACGGTGATGAGATTAACTATGTGTTCG

GGGAACCGCTCAATCCCAGCCTCGGCTACACCGAGGACGAGAAAGACTTT

AGCCGAAAGATCATGCGATACTGGTCTAACTTTGCCAAGACGGGGTATGA

TTCTTAGCGATATAACGCATTAACTATTTCTAACGATTCCATTTCCCATC

ATTTTGCCCTTCCAGCAATCCAAACCCCAACACGGCTAGTAGCGAATTTC

CCGAATGGCCGAAACATACGGCCCACGGACGGCACTATCTGGAGCTGGGC

CTCAACACGTCCTTCGTCGGACGGGGTCCCCGGTTGAGGCAGTGTGCCTT

CTGGAAGAAATATCTTCCCCAGCTAGTTGCAGCTACCTGTTAGTATCTAC

GTCACGTAAGATGTAACGCTTTCGAAACAAATAAACGTTTCTAAACCAAT

GCCTTCGTGTTCATTCGCAGCTAACATTGGAGGTGAACCACTACCAAGTG

CACCGTGCGAAAGCAGCGCATTTTTTTACCGACCTGATCTGGTTGTGCTG

CTAGTGTCA

>NIG_AL3_N485 [organism=Anopheles funestus] [Nigeria] ace-1 gDNA, partial CDS

AACAATGTATACATGTACCTGTACACGCACCGAAGCAAAGGCAACCCATG

GCCACGCTGGACGGGCGTTATGCACGGTGATGAGATTAACTATGTGTTCG

GGGAACCGCTCAATCCCAGCCTCGGCTACACCGAGGACGAGAAAGACTTT

AGCCGGAAGATCATGCGATACTGGTCAAACTTTGCCAAGACGGGGTATGA

TTATTAGCGATATAACGCATTAACTATTTCTAACGATTCCATTTCCCATC

ATTTTGCCTTTCCAGCAATCCAAACCCCAACACGGCTAGTAGCGAATTTC

CCGAATGGCCGAAACATACGGCCCACGGACGGCACTATCTGGAGCTGGGC

CTCAACACGTCCTTCGTCGGACGGGGTCCCCGGTTGAGGCAGTGTGCCTT

CTGGAAGAAATATCTTCCCCAGCTAGTTGCAGCTACCTGTTAGTATCTAC

GTCACGTAAGATGTAACGCTTTCGAAACAAATAAACGTTTCTAAACCAAT

GCCTTCGTGTTCATTCGCAGCTAACATTGGAGGTGAACCACTACCAAGTG

CACCGTGCGAAAGCAGCGCATTTTTTTACCGACCTGATCTGGTTGTGCTG

CTAGTGTCA

>NIG_AL4_N485 [organism=Anopheles funestus] [Nigeria] ace-1 gDNA, partial CDS

AACAATGTATACATGTACCTGTACACGCACCGAAGCAAAGGCAACCCATG

GCCACGCTGGACGGGCGTTATGCACGGTGATGAGATTAACTATGTGTTCG

GGGAACCGCTCAATCCCAGCCTCGGCTACACCGAGGACGAGAAAGACTTT

AGCCGGAAGATCATGCGATACTGGTCAAACTTTGCCAAGACGGGGTAGGA

TTATTAGCGATATAACGCATTAACTATTTCTAACGATTTCATTTCCCATC

ATTTTGCCTTTCCAGCAATCCAAACCCCAACACGGCTAGTAGCGAATTTC

CCGAATGGCCGAAACATACGGCCCACGGACGGCACTATCTGGAGCTGGGC

CTCAACACGTCCTTCGTCGGACGGGGTCCCCGGTTGAGGCAGTGTGCCTT

CTGGAAGAAATATCTTCCCCAGCTAGTTGCAGCTACCTGTTAGTATCTAC

GTCACGTAAGATGTAACGCTTTCGAAACAAATAAACGTTTCTAAACCAAT

GCCTTCGTGTTCATTCGCAGCTAACATTGGAGGTGAACCACTACCAAGTG

CACCGTGCGAAAGCAGCGCATTTTTTTACCGACCTGATCTGGTTGTGCTG

CTAGTGTCA

>NIG_AL5_N485 [organism=Anopheles funestus] [Nigeria] ace-1 gDNA, partial CDS

AACAATGTATACATGTACCTGTACACGCACCGAAGCAAAGGCAACCCATG

GCCACGCTGGACGGGCGTTATGCACGGTGATGAGATTAACTATGTGTTCG

GGGAACCGCTCAATCCCAGCCTCGGCTACACCGAGGACGAGAAAGACTTT

AGCCGAAAGATCATGCGATACTGGTCAAACTTTGCCAAGACGGGGTATGA

TTATTAGCGATGTAACGCATTAACTATTTCTAACGATTCCATTTCCCATC

ATTTTGCCTTTCCAGCAATCCAAACCCCAACACGGCTAGTAGCGAATTTC

CCGAATGGCCGAAACATACGGCCCACGGACGGCACTATCTGGAGCTGGGC

CTCAACACGTCCTTCGTCGGACGGGGTCCCCGGTTGAGGCAGTGTGCCTT

CTGGAAGAAATATCTTCCCCAGCTAGTTGCAGCTACCTGTTAGTATCTAC

GTCACGTAAGATGTAACGCTTTCGAAACAAATAAACGTTTCTAAACCAAT

GCCTTCGTGTTCATTCGCAGCTAACATTGGAGGTGAACCACTACCAAGTG

CACCGTGCGAAAGCAGCGCATTTTTTTACCGACCTGATCTGGTTGTGCTG

CTAGTGTCA

>NIG_AL6_N485 [organism=Anopheles funestus] [Nigeria] ace-1 gDNA, partial CDS

AACAATGTATACATGTACCTGTACACGCACCGAAGCAAAGGCAACCCATG

GCCACGCTGGACGGGCGTCATGCACGGTGATGAGATTAACTATGTGTTCG

GGGAACCGCTCAATCCCAGCCTCGGCTACACCGAGGACGAGAAAGACTTT

AGCCGGAAGATCATGCGATACTGGTCAAACTTTGCCAAGACGGGGTAGGA

TTCTTAGCAATATAACGCATTAACTATTTCTAACGATTCCATTTCCCATC

ATTTTGCCTTTCCAGCAATCCAAACCCCAACACGGCTAGTAGCGAATTTC

CCGAATGGCCGAAACATACGGCCCACGGACGGCACTATCTGGAGCTGGGC

CTCAACACGTCCTTCGTCGGACGGGGTCCCCGGTTGAGGCAGTGTGCCTT

CTGGAAGAAATATCTTCCCCAGCTAGTTGCAGCTACCTGTTAGTATCTAC

GTCACGTAAGATGTAACGCTTTCGAAACAAATAAACGTTTCTAAACCAAT

GCCTTCGTGTTCATTCGCAGCTAACATTGGAGGTGAACCACTACCAAGTG

CACCGTGCGAAAGCAGCGCATTTTTTTACCGACCTGATCTGGTTGTGCTG

CTAGTGTCA

>NIG_AL7_N485 [organism=Anopheles funestus] [Nigeria] ace-1 gDNA, partial CDS

AACAATGTATACATGTACCTGTACACGCACCGAAGCAAAGGCAACCCATG

GCCACGCTGGACGGGCGTTATGCACGGTGATGAGATTAACTATGTGTTCG

GGGAACCGCTCAATCCCAGCCTCGGCTACACCGAGGACGAGAAAGACTTT

AGCCGGAAGATCATGCGATACTGGTCAAACTTTGCCAAGACGGGGTAGGA

TTCTTAGCAATATAACGCATTAACTATTTCTAACGATTCCATTTCCCATC

ATTTTGCCTTTCCAGCAATCCAAACCCCAACACGGCTAGTAGCGAATTTC

CCGAATGGCCGAAACATACGGCCCACGGACGGCACTATCTGGAGCTGGGC

CTCAACACGTCCTTCGTCGGACGGGGTCCCCGGTTGAGGCAGTGTGCCTT

CTGGAAGAAATATCTTCCCCAGCTAGTTGCAGCTACCTGTTAGTATCTAC

GTCACGTAAGATGTAACGCTTTCGAAACAAATAAACGTTTCTAAACCAAT

GCCTTCGTGTTCATTCGCAGCTAACATTGGAGGTGAACCACTACCAAGTG

CACCGTGCGAAAGCAGCGCATTTTTTTACCGACCTGATCTGGTTGTGCTG

CTAGTGTCA

>NIG_AL8_N485 [organism=Anopheles funestus] [Nigeria] ace-1 gDNA, partial CDS

AACAATGTATACATGTACCTGTACACGCACCGAAGCAAAGGCAACCCATG

GCCACGCTGGACGGGCGTTATGCACGGTGATGAGATTAACTATGTGTTCG

GGGAACCGCTCAATCCCAGCCTCGGCTACACCGAGGACGAGAAAGACTTT

AGCCGGAAGATCATGCGATACTGGTCAAACTTTGCCAAGACGGGGTATGA

TTATTAGCGATGTAACGCATTAACTATTTCTAACGATTCCATTTCCCATC

ATTTTGCCTTTCCAGCAATCCAAACCCCAACACGGCTAGTAGCGAATTTC

CCGAATGGCCGAAACATACGGCCCACGGACGGCACTATCTGGAGCTGGGC

CTCAACACGTCCTTCGTCGGACGGGGTCCCCGGTTGAGGCAGTGTGCCTT

CTGGAAGAAATATCTTCCCCAGCTAGTTGCAGCTACCTGTTAGTATCTAC

GTCACGTAAGATGTAACGCTTTCGAAACAAATAAACGTTTCTAAACCAAT

GCCTTCGTGTTCATTCGCAGCTAACATTGGAGGTGAACCACTACCAAGTG

CACCGTGCGAAAGCAGCGCATTTTTTTACCGACCTGATCTGGTTGTGCTG

CTAGTGTCA

>NIG_AL9_N485 [organism=Anopheles funestus] [Nigeria] ace-1 gDNA, partial CDS

AACAATGTATACATGTACCTGTACACGCACCGAAGCAAAGGCAACCCATG

GCCACGCTGGACGGGCGTTATGCACGGTGATGAGATTAACTATGTGTTCG

GGGAACCGCTCAATCCCAGCCTCGGCTACACCGAGGACGAGAAAGACTTT

AGCCGAAAGATCATGCGATACTGGTCAAACTTTGCCAAGACGGGGTAGGA

TTATTAGCGATGTAACGCATTAACTATTTCTAACGATTCCATTTCCCATC

ATTTTGCCTTTCCAGCAATCCAAACCCCAACACGGCTAGTAGCGAATTTC

CCGAATGGCCGAAACATACGGCCCACGGACGGCACTATCTGGAGCTGGGC

CTCAACACGTCCTTCGTCGGACGGGGTCCCCGGTTGAGGCAGTGTGCCTT

CTGGAAGAAATATCTTCCCCAGCTAGTTGCAGCTACCTGTTAGTATCTAC

GTCACGTAAGATGTAACGCTTTCGAAACAAATAAACGTTTCTAAACCAAT

GCCTTCGTGTTCATTCGCAGCTAACATTGGAGGTGAACCACTACCAAGTG

CACCGTGCGAAAGCAGCGCATTTTTTTACCGACCTGATCTGGTTGTGCTG

CTAGTGTCA

>NIG_AL10_N485 [organism=Anopheles funestus] [Nigeria] ace-1 gDNA, partial CDS

AACAATGTATACATGTACCTGTACACGCACCGAAGCAAAGGCAACCCATG

GCCACGCTGGACGGGCGTTATGCACGGTGATGAGATTAACTATGTGTTCG

GGGAACCGCTCAATCCCAGCCTCGGCTACACCGAGGACGAGAAAGACTTT

AGCCGAAAGATCATGCGATACTGGTCCAACTTTGCCAAGACGGGGTAGGA

TTATTAGCGATGTAACGCATTAACTATTTCTAACGATTCCATTTCCCATC

ATTTTGCCTTTCCAGCAATCCAAACCCCAACACGGCTAGTAGCGAATTTC

CCGAATGGCCGAAACATACGGCCCACGGACGGCACTATCTGGAGCTGGGC

CTCAACACGTCCTTCGTCGGACGGGGTCCCCGGTTGAGGCAATGTGCCTT

CTGGAAGAAATATCTTCCCCAGCTAGTTGCAGCTACCTGTTAGTATCTAC

GTCACGTAAGATGTAACGCTTTCGAAACAAATAAACGTTTCTAAACCAAT

GCCTTCGTGTTCATTCGCAGCTAACATTGGAGGTGAACCACTACCAAGTG

CACCGTGCGAAAGCAGCGCATTTTTTTACCGACCTGATCTGGTTGTGCTG

CTAGTGTCA

>NIG_AL11_N485 [organism=Anopheles funestus] [Nigeria] ace-1 gDNA, partial CDS

AACAATGTATACATGTACCTGTACACGCACCGAAGCAAAGGCAACCCATG

GCCACGCTGGACGGGCGTTATGCACGGTGATGAGATTAACTATGTGTTCG

GGGAACCGCTCAATCCCAGCCTCGGCTACACCGAGGACGAGAAAGACTTT

AGCCGAAAGATCATGCGATACTGGTCAAACTTTGCCAAGACGGGGTATGA

TTATTAGCGATGTAACGCATTAACTATTTCTAACGATTCCATTTCCCATC

ATTTTGCCCTTCCAGCAATCCAAACCCCAACACGGCTAGTAGCGAATTTC

CCGAATGGCCGAAACATACGGCCCACGGACGGCACTATCTGGAGCTGGGC

CTCAACACGTCCTTCGTCGGACGGGGTCCCCGGTTGAGGCAGTGTGCCTT

CTGGAAGAAATATCTTCCCCAGCTAGTTGCAGCTACCTGTTAGTATCTAC

GTCACGTAAGATGTAACGCTTTCGAAACAAATAAACGTTTCTAAACCAAT

GCCTTCGTGTTCATTCGCAGCTAACATTGGAGGTGAACCACTACCAAGTG

CACCGTGCGAAAGCAGCGCATTTTTTTACCGACCTGATCTGGTTGTCGTG

TCTATGTCA

>NIG_AL12_N485 [organism=Anopheles funestus] [Nigeria] ace-1 gDNA, partial CDS

AACAATGTATACATGTACCTGTACACGCACCGAAGCAAAGGCAACCCATG

GCCACGCTGGACGGGCGTTATGCACGGTGATGAGATTAACTATGTGTTCG

GGGAACCGCTCAATCCCAGCCTCGGCTACACCGAGGACGAGAAAGACTTT

AGCCGGAAGATCATGCGATACTGGTCAAACTTTGCCAAGACGGGGTAGGA

TTCTTAGCAATATAACGCATTAACTATTTCTAACGATTCCATTTCCCATC

ATTTTGCCTTTCCAGCAATCCAAACCCCAACACGGCTAGTAGCGAATTTC

CCGAATGGCCGAAACATACGGCCCACGGACGGCACTATCTGGAGCTGGGC

CTCAACACGTCCTTCGTCGGACGGGGTCCCCGGTTGAGGCAGTGTGCCTT

CTGGAAGAAATATCTTCCCCAGCTAGTTGCAGCTACCTGTTAGTATCTAC

GTCACGTAAGATGTAACGCTTTCGAAACAAATAAACGTTTCTAAACCAAT

GCCTTCGTGTTCATTCGCAGCTAACATTGGAGGTGAACCACTACCAAGTG

CACCGTGCGAAAGCAGCGCATTTTTTTACCGACCTGATCTGGTGGTGCTG

CTAGTGTCA

>NIG_DE1_N485 [organism=Anopheles funestus] [Nigeria] ace-1 gDNA, partial CDS

AACAATGTATACATGTACCTGTACACGCACCGAAGCAAAGGCAACCCATG

GCCACGCTGGACGGGCGTTATGCACGGTGATGAGATTAACTATGTGTTCG

GGGAACCGCTCAATCCCAGCCTCGGCTACACCGAGGACGAGAAAGACTTT

AGCCGGAAGATCATGCGATACTGGTCAAACTTTGCCAAGACGGGGTAGGA

TTCTTAGCAATATAACGCAATAACTATTTCTAACGATTCCATTTCCCATC

ATTTTGCCTTTCCAGCAATCCAAACCCCAACACGGCTAGTAGCGAATTTC

CCGAATGGCCGAAACATACGGCCCACGGACGGCACTATCTGGAGCTGGGC

CTCAACACGTCCTTCGTCGGACGGGGTCCCCGGTTGAGGCAGTGTGCCTT

CTGGAAGAAATATCTTCCCCAGCTAGTTGCAGCTACCTGTTAGTATCTAC

GTCACGTAAGATGTAACGCTTTCGAAACAAATAAACGTTTCTAAACCAAT

GCCTTCGTGTTCATTCGCAGCTAACATTGGAGGTGAACCACTACCAAGTG

CACCGTGCGAAAGCAGCGCATTTTTTTACCGACCTGATCTGGTTGTGCTG

CTAGTGTCA

>NIG_DE2_N485 [organism=Anopheles funestus] [Nigeria] ace-1 gDNA, partial CDS

AACAATGTATACATGTACCTGTACACGCACCGAAGCAAAGGCAACCCATG

GCCACGCTGGACGGGCGTTATGCACGGTGATGAGATTAATTATGTGTTCG

GGGAACCGCTGAATCCCAGCCTCGGCTACACCGAGGACGAGAAAGACTTT

AGCCGAAAGATCATGCGATACTGGTCAAACTTTGCCAAGACGGGGTAGGA

TTATTAGCGATGTAACGCATTAACTATTTCTAACGATTCCATTTCCCATC

ATTTTGCCTTTCCAGCAATCCAAACCCCAACACGGCTAGTAGCGAATTTC

CCGAATGGCCGAAACATACGGCCCACGGACGGCACTATCTGGAGCTGGGC

CTCAACACGTCCTTCGTCGGACGGGGTCCCCGGTTGAGGCAGTGTGCCTT

CTGGAAGAAATATCTTCCCCAGCTAGTTGCAGCTACCTGTTAGTATCTAC

GTCACGTAAGATGTAACGCTTTCGAAACAAATAAACGTTTCTAAACCAAT

GCCTTCGTGTTCATTCGCAGCTAACATTGGAGGTGAACCACTACCAAGTG

CACCGTGCGAAAGCAGCGCATTTTTTTACCGACCTGATCTGGTGGTGCTG

CTAGTGTCA

>NIG_DE3_N485 [organism=Anopheles funestus] [Nigeria] ace-1 gDNA, partial CDS

AACAATGTATACATGTACCTGTACACGCACCGAAGCAAAGGCAACCCATG

GCCACGCTGGACGGGCGTTATGCACGGTGATGAGATTAACTATGTGTTCG

GGGAACCGCTCAATCCCAGCCTCGGCTACACCGAGGACGAGAAAGACTTT

AGCCGAAAGATCATGCGATACTGGTCAAACTTTGCCAAGACGGGGTAGGA

TTATTAGCAATATAACGCATTAACTATTTCTAACGATTCCATTTCCCATC

ATTTTGCCTTTCCAGCAATCCAAACCCCAACACGGCTAGTAGCGAATTTC

CCGAATGGCCGAAACATACGGCCCACGGACGGCACTATCTGGAGCTGGGC

CTCAACACGTCCTTCGTCGGACGGGGTCCCCGGTTGAGGCAGTGTGCCTT

CTGGAAGAAATATCTTCCCCAGCTAGTTGCAGCTACCTGTTAGTATCTAC

GTCACGTAAGATGTAACGCTTTCGAAACAAATAAACGTTTCTAAACCAAT

GCCTTCGTGTTCATTCGCAGCTAACATTGGAGGTGAACCACTACCAAGTG

CACCGTGCGAAAGCAGCGCATTTTTTTACCGACCTGATCTGGTTGTGCTG

CTAGTGTCA

>NIG_DE4_N485 [organism=Anopheles funestus] [Nigeria] ace-1 gDNA, partial CDS

AACAATGTATACATGTACCTGTACACGCACCGAAGCAAAGGCAACCCATG

GCCACGCTGGACGGGCGTTATGCACGGTGATGAGATTAACTATGTGTTCG

GGGAACCGCTGAATCCCAGCCTCGGCTACACCGAGGACGAGAAAGACTTT

AGCCGAAAGATCATGCGATACTGGTCCAACTTTGCCAAGACGGGGTAGGA

TTATTAGCGATGTAACGCATTAACTATTTCTAACGATTCCATTTCCCATC

ATTTTGCCCTTCCAGCAATCCAAACCCCAACACGGCTAGTAGCGAATTTC

CCGAATGGCCGAAACATACGGCCCACGGACGGCACTATCTGGAGCTGGGC

CTCAACACGTCCTTCGTCGGACGGGGTCCCCGGTTGAGGCAATGTGCCTT

CTGGAAGAAATATCTTCCCCAGCTAGTTGCAGCTACCTGTTAGTATCTAC

GTCACGTAAGATGTAACGCTTTCGAAACAAATAAACGTTTCTAAACCAAT

GCCTTCGTGTTCATTCGCAGCTAACATTGGAGGTGAACCACTACCAAGTG

CACCGTGCGAAAGCAGCGCATTTTTTTACCGACCTGATCTGGTGTTGCTG

CTAGTGTCA

>NIG_DE5_N485 [organism=Anopheles funestus] [Nigeria] ace-1 gDNA, partial CDS

AACAATGTATACATGTACCTGTACACGCACCGAAGCAAAGGCAACCCATG

GCCACGCTGGACGGGCGTTATGCACGGTGATGAGATTAACTATGTGTTCG

GGGAACCGCTCAATCCCAGCCTCGGCTACACCGAGGACGAGAAAGACTTT

AGCCGAAAGATCATGCGATACTGGTCAAACTTTGCCAAGACGGGGTAGGA

TTCTTAGCAATATAACGCATTAACTATTTCTAACGATTCCATTTCCCATC

ATTTTGCCTTTCCAGCAATCCAAACCCCAACACGGCTAGTAGCGAATTTC

CCGAATGGCCGAAACATACGGCCCACGGACGGCACTATCTGGAGCTGGGC

CTCAACACGTCCTTCGTCGGACGGGGTCCCCGGTTGAGGCAGTGTGCCTT

CTGGAAGAAATATCTTCCCCAGCTAGTTGCAGCTACCTGTTAGTATCTAC

GTCACGTAAGATGTAACGCTTTCGAAACAAATAAACGTTTCTAAACCAAT

GCCTTCGTGTTCATTCGCAGCTAACATTGGAGGTGAACCACTACCAAGTG

CACCGTGCGAAAGCAGCGCATTTTTTTACCGACCTGATCTGGTTGTGCTG

CTAGTGTCA

>NIG_DE6_N485 [organism=Anopheles funestus] [Nigeria] ace-1 gDNA, partial CDS

AACAATGTATACATGTACCTGTACACGCACCGAAGCAAAGGCAACCCATG

GCCACGCTGGACGGGCGTCATGCACGGTGATGAGATTAACTATGTGTTCG

GGGAACCGCTCAATCCCAGCCTCGGCTACACCGAGGACGAGAAAGACTTT

AGCCGAAAGATCATGCGATACTGGTCAAACTTTGCCAAGACGGGGTAGGA

TTCTTAGCAATATAACGCATTAACTATTTCTAACGATTCCATTTCCCATC

ATTTTGCCTTTCCAGCAATCCAAACCCCAACACGGCTAGTAGCGAATTTC

CCGAATGGCCGAAACATACGGCCCACGGACGGCACTATCTGGAGCTGGGC

CTCAACACGTCCTTCGTCGGACGGGGTCCCCGGTTGAGGCAGTGTGCCTT

CTGGAAGAAATATCTTCCCCAGCTAGTTGCAGCTACCTGTTAGTATCTAC

GTCACGTAAGATGTAACGCTTTCGAAACAAATAAACGTTTCTAAACCAAT

GCCTTCGTGTTCATTCGCAGCTAACATTGGAGGTGAACCACTACCAAGTG

CACCGTGCGAAAGCAGCGCATTTTTTTACCGACCTGATCTGGTTGTGCTG

CTAGTGTCA

>NIG_DE7_N485 [organism=Anopheles funestus] [Nigeria] ace-1 gDNA, partial CDS

AACAATGTATACATGTACCTGTACACGCACCGAAGCAAAGGCAACCCATG

GCCACGCTGGACGGGCGTTATGCACGGTGATGAGATTAACTATGTGTTCG

GGGAACCGCTCAATCCCAGCCTCGGCTACACCGAGGACGAGAAAGACTTT

AGCCGAAAGATCATGCGATACTGGTCAAACTTTGCCAAGACGGGGTATGA

TTATTAGCGATGTAACGCATTAACTATTTCTAACGATTCCATTTCCCATC

ATTTTGCCTTTCCAGCAATCCAAACCCCAACACGGCTAGTAGCGAATTTC

CCGAATGGCCGAAACATACGGCCCACGGACGGCACTATCTGGAGCTGGGC

CTCAACACGTCCTTCGTCGGACGGGGTCCCCGGTTGAGGCAGTGTGCCTT

CTGGAAGAAATATCTTCCCCAGCTAGTTGCAGCTACCTGTTAGTATCTAC

GTCACGTAAGATGTAACGCTTTCGAAACAAATAAACGTTTCTAAACCAAT

GCCTTCGTGTTCATTCGCAGCTAACATTGGAGGTGAACCACTACCAAGTG

CACCGTGCGAAAGCAGCGCATTTTTTTACCGACCTGATCTGGTTGTGCTG

CTAGTGTCA

>NIG_DE8_N485 [organism=Anopheles funestus] [Nigeria] ace-1 gDNA, partial CDS

AACAATGTATACATGTACCTGTACACGCACCGAAGCAAAGGCAACCCATG

GCCACGCTGGACGGGCGTTATGCACGGTGATGAGATTAACTATGTGTTCG

GGGAACCGCTCAATCCCAGCCTCGGCTACACCGAGGACGAGAAAGACTTT

AGCCGAAAGATCATGCGATACTGGTCAAACTTTGCCAAGACGGGGTAGGA

TTCTTAGCAATATAACGCATTAACTATTTCTAACGATTCCATTTCCCATC

ATTTTGCCTTTCCAGCAATCCAAACCCCAACACGGCTAGTAGCGAATTTC

CCGAATGGCCGAAACATACGGCCCACGGACGGCACTATCTGGAGCTGGGC

CTCAACACGTCCTTCGTCGGACGGGGTCCCCGGTTGAGGCAGTGTGCCTT

CTGGAAGAAATATCTTCCCCAGCTAGTTGCAGCTACCTGTTAGTATCTAC

GTCACGTAAGATGTAACGCTTTCGAAACAAATAAACGTTTCTAAACCAAT

GCCTTCGTGTTCATTCGCAGCTAACATTGGAGGTGAACCACTACCAAGTG

CACCGTGCGAAAGCAGCGCATTTTTTTACCGACCTGATCTGGTGGTGCTG

CTAGTGTCA

>NIG_DE9_N485 [organism=Anopheles funestus] [Nigeria] ace-1 gDNA, partial CDS

AACAATGTATACATGTACCTGTACACGCACCGAAGCAAAGGCAACCCATG

GCCACGCTGGACGGGCGTTATGCACGGTGATGAGATTAACTATGTGTTCG

GGGAACCGCTCAATCCCAGCCTCGGCTACACCGAGGACGAGAAAGACTTT

AGCCGAAAGATCATGCGATACTGGTCAAACTTTGCCAAGACGGGGTAGGA

TTCTTAGCAATATAACGCATTAACTATTTCTAACGATTCCATTTCCCATC

ATTTTGCCTTTCCAGCAATCCAAACCCCAACACGGCTAGTAGCGAATTTC

CCGAATGGCCGAAACATACGGCCCACGGACGGCACTATCTGGAGCTGGGC

CTCAACACGTCCTTCGTCGGACGGGGTCCCCGGTTGAGGCAGTGTGCCTT

CTGGAAGAAATATCTTCCCCAGCTAGTTGCAGCTACCTGTTAGTATCTAC

GTCACGTAAGATGTAACGCTTTCGAAACAAATAAACGTTTCTAAACCAAT

GCCTTCGTGTTCATTCGCAGCTAACATTGGAGGTGAACCACTACCAAGTG

CACCGTGCGAAAACAGCGCATTTTTTTACCGACCTGATCTGGTTGTGCTG

CTAGTGTCA

>NIG_DE10_N485 [organism=Anopheles funestus] [Nigeria] ace-1 gDNA, partial CDS

AACAATGTATACATGTACCTGTACACGCACCGAAGCAAAGGCAACCCATG

GCCACGCTGGACGGGCGTTATGCACGGTGATGAGATTAACTATGTGTTCG

GGGAACCGCTCAATCCCAGCCTCGGCTACACCGAGGACGAGAAAGACTTT

AGCCGAAAGATCATGCGATACTGGTCAAACTTTGCCAAGACGGGGTAGGA

TTATTAGCGATGTAACGCATTAACTATTTCTAACGATTCCATTTCCCATC

ATTTTGCCTTTCCAGCAATCCAAACCCCAACACGGCTAGTAGCGAATTTC

CCGAATGGCCGAAACATACGGCCCACGGACGGCACTATCTGGAGCTGGGC

CTCAACACGTCCTTCGTCGGACGGGGTCCCCGGTTGAGGCAGTGTGCCTT

CTGGAAGAAATATCTTCCCCAGCTAGTTGCAGCTACCTGTTAGTATCTAC

GTCACGTAAGATGTAACGCTTTCGAAACAAATAAACGTTTCTAAACCAAT

GCCTTCGTGTTCATTCGCAGCTAACATTGGAGGTGAACCACTACCAAGTG

CACCGTGCGAAAGCAGCGCATTTTTTTACCGACCTGATCTGGTGGTGCTG

CTAGTGTCA

>NIG_DE11_N485 [organism=Anopheles funestus] [Nigeria] ace-1 gDNA, partial CDS

AACAATGTATACATGTACCTGTACACGCACCGAAGCAAAGGCAACCCATG

GCCACGCTGGACGGGCGTTATGCACGGTGATGAGATTAACTATGTGTTCG

GGGAACCGCTCAATCCCAGCCTCGGCTACACCGAGGACGAGAAAGACTTT

AGCCGAAAGATCATGCGATACTGGTCAAACTTTGCCAAGACGGGGTAGGA

TTCTTAGCAATATAACGCATTAACTATTTCTAACGATTCCATTTCCCATC

ATTTTGCCCTTCCAGCAATCCAAACCCCAACACGGCTAGTAGCGAATTTC

CCGAATGGCCGAAACATACGGCCCACGGACGGCACTATCTGGAGCTGGGC

CTCAACACGTCCTTCGTCGGACGGGGTCCCCGGTTGAGGCAGTGTGCCTT

CTGGAAGAAATATCTTCCCCAGCTAGTTGCAGCTACCTGTTAGTATCTAC

GTCACGTAAGATGTAACGCTTTCGAAACAAATAAACGTTTCTAAACCAAT

GCCTTCGTGTTCATTCGCAGCTAACATTGGAGGTGAACCACTACCAAGTG

CACCGTGCGAAAGCAGCGCATTTTTTTACCGACCTGATCTGGTTGTGCTG

CTAGTGTCA

>NIG_DE12_N485 [organism=Anopheles funestus] [Nigeria] ace-1 gDNA, partial CDS

AACAATGTATACATGTACCTGTACACGCACCGAAGCAAAGGCAACCCATG

GCCACGCTGGACGGGCGTTATGCACGGTGATGAGATTAACTACGTGTTCG

GGGAACCGCTCAATCCCAGCCTCGGCTACACCGAGGACGAGAAAGACTTT

AGCCGAAAGATCATGCGATACTGGTCAAACTTTGCCAAGACGGGGTAGGA

TTCTTAGCAATATAACGCATTAACTATTTCTAACGATTCCATTTCCCATC

ATTTTGCCTTTCCAGCAATCCAAACCCCAACACGGCTAGTAGCGAATTTC

CCGAATGGCCGAAACATACGGCCCACGGACGGCACTATCTGGAGCTGGGC

CTCAACACGTCCTTCGTCGGACGGGGTCCCCGGTTGAGGCAGTGTGCCTT

CTGGAAGAAATATCTTCCCCAGCTAGTTGCAGCTACCTGTTAGTATCTAC

GTCACGTAAGATGTAACGCTTTCGAAACAAATAAACGTTTCTAAACCAAT

GCCTTCGTGTTCATTCGCAGCTAACATTGGAGGTGAACCACTACCAAGTG

CACCGTGCGAAAGCAGCGCATTTTTTTACCGACCTGATCTGGTGGTGCTG

CTAGTGTCA

Nigeria *An. funestus* voltage-gated sodium channel L1014 partial gDNA sequences

>NIG1_Afunvgsc [organism=Anopheles funestus] [Nigeria] vgsc gDNA, partial CDS

GTTCAATGAAGCCCCTCAAATGGTTGATACATTCGTAAAAAGACTAGAAA

CATAATTCGAAAGACTAGAGATGAAACAATGAATAAGACTGAAGGTAGAG

TATTTAGCGTACAATATCCATTGTTTCTTAAACATATTGCTTTATGTTCA

AATATGCGCCTTGCAAATCAATTACAATTTATAACTTTTTATCATTTGTA

AGAAAATCTATGACTGCTGATGTGTCACCTAAATTATTTACAATTGTGTC

GTTATTTGTTATCTGATCACTAAACACAAAGGTTTAAGGTCGATGCATCC

GTATAAATTTGTGTAAAACTACAATAATTTCATTTGAGTGCCAGAGACTT

CAACGATCGTATTTAAATCAGTCGCTATTTTCTTGCATTTGGTATTTTGT

TCTGATTTACAGCGAACATGTACTAGACTGTTCAATGTCCGCTGGTTACT

GTCTTGCATACCATTGTTTGCTAAACAAAACAAAATAACAACAAATTAAA

CGACAAGCTTTTAGAACAACAAGAGCTCTAGAAACAGCTTACATATGCCT

CGATAATCTGCAATCTGCTAGCTTCTCCACGCGGATTATTTGTAAATCAA

GTTCATTCACATTTTTGTCAGAGTGCTGGTGAACGCCTAATCCTAATCGC

GCATCGTTCGTATTTTTCTTCAATTCTATTCGAAATAAATTCTTCCAGAT

AATGTGCATCTGTTCCCAGACCAAGATCTGCCAAGATGGAATTTTACCGA

TTTCATGCATTCCTTTATGATTGTGTTCCGTGTGCTGTGCGGTGAATGGA

TCGAATCCATGTGGGACTGTATGCTTGTTGGCGATGTGTCATGCATACCA

TTTTTCCTGGCTACGGTAGTAATAGGAAATTTAGTCGTAAGTAACCTAGC

ATTTGCA

>NIG2_Afunvgsc [organism=Anopheles funestus] [Nigeria] vgsc gDNA, partial CDS

GTTCAATGAAGCCCCTCAAATGGTTGATACATTCGTAAAAAGACTAGAAA

CATAATTCGAAAGACTAGAGATGAAACAATGAATAAGACTGAAGGTAGAG

TATTTAGCGTACAATATCCATTGTTTCTTAAACATATTGCTTTATGTTCA

AATATGCGCCTTGCAAATCAATTACAATTTATAACTTTTTATCATTTGTA

AGAAAATCTATGACTGCTGATGTGTCACCTAAATTATTTACAATTGTGTC

GTTATTTGTTATCTGATCACTAAACTCAAAGGTTTAAGGTCGATGCATCC

GTATAAATTTGTGTAAAACTACAATAATTTCATTTGAGTGCCAGAGACTT

CAACGATCGTATTTAACTCAGTCGCTATTTTCTTGCATTTGGTATTTTGT

TCTGATTTACAGCGAACATGTACTAGACTGTTCAATGTCCGCTGGTTACT

GTCTTGCATACCATTGTTTGCTAAACAAAACAAAATAACAACAAATTAAA

CGACAAGCTTTTAGAACAACAAGAGCTCTAGAAACAGCTTACATATGCCT

CGATAATCTGCAATCTGCTAGCTTCTCCACGCGGATTATTTGTAAATCAA

GTGCATTCACATTTTTCTCAGAGTGCTGGTGAACGCCTAATCCTAATCGC

GCATCGTTCGTATTTTTCTTCAATTCTATTCGAAATAAATTCTTCCAGAT

AATGTGCATCTGTTCCCAGACCAAGATCTGCCAAGATGGAATTTTACCGA

TTTCATGCATTCCTTTATGATTGTGTTCCGTGTGCTGTGCGGTGAATGGA

TCGAATCCATGTGGGACTGTATGCTTGTTGGCGATGTGTCATGCATACCA

TTTTTCCTGGCTACGGTAGTAATAGGAAATTTAGTCGTAAGTAACCTAGC

ATTTGCA

>NIG3_Afunvgsc [organism=Anopheles funestus] [Nigeria] vgsc gDNA, partial CDS

GTTCAATGAAGCCCCTCAAATGGTTGATACATTCGTAAAAAGACTAGAAA

CATAATTCGAAAGACTAGAGATGAAACAATGAATAAGACTGAAGGTAGAG

TATTTAGCGTACAATATCCATTGTTTCTTAAACATATTGCTTTATGTTCA

AATATGCGCCTTGCAAATCAATTACAATTTATAACTTTTTATCATTTGTA

AGAAAATCTATGACTGCTAATGTGTCACCTAAATTATTTACAATTGTGTC

GTTATTTGTTATCTGATCACTAAACACAAAGGTTTAAGGTCGATGCATCC

GTATAAATTTGTGTAAAACTACAATAATTTCATTTGAGTGCCAGAGACTT

CAACGATCGTATTTAAATCAGTCGCTATTTTCTTGCATTTGGTATTTTGT

TCTGATTTACAGCGAACATGTACTAGACTGTTCAATGTCCGCTGGTTACT

GTCTTGCATACCATTGTTTGCTAAACAAAACAAAATAACAACAAATTAAA

CGACAAGCTTTTAGAACAACAAGAGCTCTAGAAACAGCTTACATATGCCT

CGATAATCTGCAATCTGCTAGCTTCTCCACGCGGATTATTTGTAAATCAA

GTGCATTCACATTGTTCTTAGAGTGCTGGTGAACGCCTAATCCTAATCGC

GCATCGTTCGTATTTTTCTTCAATTCTATTCGAAATAAATTCTTCCAGAT

AATGTGCATCTGTTCCCAGACCAAGATCTGCCAAGATGGAATTTTACCGA

TTTCATGCATTCCTTTATGATTGTGTTCCGTGTGCTGTGCGGTGAATGGA

TCGAATCCATGTGGGACTGTATGCTTGTTGGCGATGTGTCATGCATACCA

TTTTTCCTGGCTACGGTAGTAATAGGAAATTTAGTCGTAAGTAACCTAGC

ATTTGCA

>NIG4_Afunvgsc [organism=Anopheles funestus] [Nigeria] vgsc gDNA, partial CDS

GTTCAAAGGAACCCTTCAAATGGTTGATACATTCGTAAAAAGACTAGAAA

CATAATTCGAAAGACTAGAGATGAAACAATGAATAAGACTGAAGGTAGAG

TATTTAGCGTACAATATCCATTGTTTCTTAAACATATTGCTTTATGTTCA

AATATGCGCCTTGCAAATCAATTACAATTTATAACTTTTTATCATTTGTA

AGAAAATCTATGACTGCTGATGTGTCACCTAAATTATTTACAATTGTGTC

GTTAATTGTTATCTGATCACTAAACACAAAGGTTTAAGGTCGATGCATCC

GTATAAATTTGTGTAAAACTACAATAATTTCATTTGAGTGCCAGAGACTT

CAACGATCGTATTTAAATCAGTCGCTATTTTCTTGCATTTGGTATTTTGT

TCTGATTTACAGCGAACATGTACTAGACTGTTCAATGTCCGCTGGTTACT

GTCTTGCATACCATTGTTTGCTAAACAAAACAAAATAACAACAAATTAAA

CGACAAGCTTTTAGAACAACAAGAGCTCTAGAAACAGCTTACATATGCCT

CGATAATCTGCAATCTGCTAGCTTCTCCACGCGGATTATTTGTAAATCAA

GTGCATTCACATTTTTCTCAGAGTGCTGGTGAACGCCTAATCCTAATCGC

GCATCGTTCGTATTTTTCTTCAATTCTATTCGAAATAAATTCTTCCAGAT

AATGTGCATCTGTTCCCAGACCAAGATCTGCCAAGATGGAATTTTACCGA

TTTCATGCATTCCTTTATGATTGTGTTCCGTGTGCTGTGCGGTGAATGGA

TCGAATCCATGTGGGACTGTATGCTTGTTGGCGATGTGTCATGCATACCA

TTTTTCCTGGCTACGGTAGTAATAGGAAATTTAGTCGTAAGTAACCTAGC

ATTTGCA

>NIG5_Afunvgsc [organism=Anopheles funestus] [Nigeria] vgsc gDNA, partial CDS

GTTCAATGAAGCCCCTCAAATGGTTGATACATTCGTAAAAAGACTAGAAA

CATAATTCGAAAGACTAGAGATGAAACAATGAATAAGACTGAAGGTAGAG

TATTTAGCGTACAATATCCATTGTTTCTTAAACATATTGCTTTATGTTCA

AATATGCGCCTTGCAAATCAATTACAATTTATAACTTTTTATCATTTGTA

AGAAAATCTATGACTGCTGATGTGTCACCTAAATTATTTACAATTGTGTC

GTTATTTGTTATCTGATCACTAAACACAAAGGTTTAAGGTCGATGCATCC

GTATAAATTTGTGTAAAACTACAATAATTTCATTTGAGTGCCAGAGACTT

CAACGATCGTATTTAAATCAGTCGCTATTTTCTTGCATTTGGTATTTTGT

TCTGATTTACAGCGAACATGTACTAGACTGTTCAATGTCCGCTGGTTACT

GTCTTGCATACCATTGTTTGCTAAACAAAACAAAATAACAACAAATTAAA

CGACAAGCTTTTAGAACAACAAGAGCTCTAGAAACAGCTTACATATGCCT

CGATAATCTGCAATCTGCTAGCTTCTCCACGCGGATTATTTGTAAATCAA

GTGCATTCACATTTTTCTCAGAGTGCTGGTGAACGCCTAATCCTAATCGC

GCATCGTTCGTATTTTTCTTCAATTCTATTCGAAATAAATTCTTCCAGAT

AATGTGCATCTGTTCCCAGACCAAGATCTGCCAAGATGGAATTTTACCGA

TTTCATGCATTCCTTTATGATTGTGTTCCGTGTGCTGTGCGGTGAATGGA

TCGAATCCATGTGGGACTGTATGCTTGTTGGCGATGTGTCATGCATACCA

TTTTTCCTGGCTACGGTAGTAATAGGAAATTTAGTCGTAAGTAACCTAGC

ATTTGCA

>NIG6_Afunvgsc [organism=Anopheles funestus] [Nigeria] vgsc gDNA, partial CDS

GTTCAAGGAAGCCCCCCAAAAGGTTGAGTCATTCGTAAAAAGGCTAGAAA

CATAATTCGAAAGACTAGAGATGAACCAATGAATAAGACTGAAGGTAGAG

TATTTAGCGTACAATATCCATTGTTCCTTAAACATATTGCTTTATGTTCA

AATATGCGCCTTGCAAATCAATTACAATTTATAACTTTTTATCATTTGTA

AGAAAATCTATGACTGCTAATGTGTCACCTAAATTATTTACAATTGTGTC

GTTATTTGTTATCTGATCATTAAACACAAAGGTTTAAGGTCGATGCATCC

GTATAAATTTGTGTAAAACTACAATAATTTCATTTGAGTGCCAGAGACTT

CAACGATCGTATTTAAATCAGTCGCTATTTTCTTGCATTTGGTATTTTGT

TCTGATTTACAGCGAACATGTACTAGACTGTTCAATGTCCGCTGGTTACT

GTCTTGCATACCATTGTTTGCTAAACAAAACAAAATAACAACAAATTAAA

CGACAAGCTTTTAGAACAACAAGAGCTCTAGAAACAGCTTACATATGCCT

CGATAATCTGCAATCTGCTAGCTTCTCCACGCGGATTATTTGTAAATCAA

GTGCATTCACATTTTTCTTAGAGTGCTGGTGAACGCCTAATCCTAATCGC

GCATCGTTCGTATTTTTCTTCAATTCTATTCGAAATAAATTCTTCCAGAT

AATGTGCATCTGTTCCCAGACCAAGATCTGCCAAGATGGAATTTTACCGA

TTTCATGCATTCCTTTATGATTGTGTTCCGTGTGCTGTGCGGTGAATGGA

TCGAATCCATGTGGGACTGTATGCTTGTTGGCGATGTGTCATGCATACCA

TTTTTCCTGGCTACGGTAGTAATAGGAAATTTAGTCGTAAGTAACCTAGC

ATTTGCA

>NIG7_Afunvgsc [organism=Anopheles funestus] [Nigeria] vgsc gDNA, partial CDS

GTTTAATAAAGCCCCTCAAATGGTTGATACATTCGTAAAAAGACTAGAAA

CATAATTCGAAAGACTAGAGATGAAACAATGAATAAGACTGAAGGTAGAG

TATTTAGCGTACAATATCCATTGTTTCTTAAACATATTGCTTTATGTTCA

AATATGCGCCTTGCAAATCAATTACAATTTATAACTTTTTATCATTTGTA

AGAAAATCTATGACTGCTGATGTGTCACCTAAATTATTTACAATTGTGTC

GTTATTTGTTATCTGATCACTAAACACAAAGGTTTAAGGTCGATGCATCC

GTATAAATTTGTGTAAAACTACAATAATTTCATTTGAGTTGCAGAGACTT

CAACGATCGTATTTAAATCAGTCGCTATTTTCTTGCATTTGGTATTTTGT

TCTGATTTACAGCGAACATGTACTAGACTGTTCAATGTCCGCTGGTTACT

GTCTTGCATACCATTGTTTGCTAAACAAAACAAAATAACAACAAATTAAA

CGACAAGCTTTTAGAACAACAAGAGCTCTAGAAACAGCTTACATATGCCT

CGATAATCTGCAATCTGCTAGCTTCTCCACGCGGATTATTTGTAAATCAA

GTGCATTCACATTGTTCTTAGAGTGCTGGTGAACGCCTAATCCTAATCGC

GCATCGTTCGTATTTTTCTTCAATTCTATTCGAAATAAATTCTTCCAGAT

AATGTGCATCTGTTCCCAGACCAAGATCTGCCAAGATGGAATTTTACCGA

TTTCATGCATTCCTTTATGATTGTGTTCCGTGTGCTGTGCGGTGAATGGA

TCGAATCCATGTGGGACTGTATGCTTGTTGGCGATGTGTCATGCATACCA

TTTTTCCTGGCTACGGTAGTAATAGGAAATTTAGTCGTAAGTAACCTAGC

ATTTGCA

>NIG8_Afunvgsc [organism=Anopheles funestus] [Nigeria] vgsc gDNA, partial CDS

GTTCAATGAAGCCCCTCAAATGGTTGATACATTCGTAAAAAGACTAGAAA

CATAATTCGAAAGACTAGAGATGAAACAATGAATAAGACTGAAGGTAGAG

TATTTAGCGTACAATATCCATTGTTTCTTAAACATATTGCTTTATGTTCA

AATATGCGCCTTGCAAATCAATTACAATTTATAACTTTTTATCATTTGTA

AGAAAATCTATGACTGCTGATGTGTCACCTAAATTATTTACAATTGTGTC

GTTATTTGTTATCTGATCACTAAACACAAAGGTTTAAGGTCGATGCATCC

GTATAAATTTGTGTAAAACTACAATAATTTCATTTGAGTGCCAGAGACTT

CAACGATCGTATTTAAATCAGTCGCTATTTTCTTGCATTTGGTATTTTGT

TCTGATTTACAGCGAACATGTACTAGACTGTTCAATGTCCGCTGGTTACT

GTCTTGCATACCATTGTTTGCTAAACAAAACAAAATAACAACAAATTAAA

CGACAAGCTTTTAGAACAACAAGAGCTCTAGAAACAGCTTACATATGCCT

CGATAATCTGCAATCTGCTAGCTTCTCCACGCGGATTATTTGTAAATCAA

GTGCATTCACATTTTTCTTAGAGTGCTGGTGAACGCCTAATCCTAATCGC

GCATCGTTCGTATTTTTCTTCAATTCTATTCGAAATAAATTCTTCCAGAT

AATGTGCATCTGTTCCCAGACCAAGATCTGCCAAGATGGAATTTTACCGA

TTTCATGCATTCCTTTATGATTGTGTTCCGTGTGCTGTGCGGTGAATGGA

TCGAATCCATGTGGGACTGTATGCTTGTTGGCGATGTGTCATGCATACCA

TTTTTCCTGGCTACGGTAGTAATAGGAAATTTAGTCGTAAGTAACCTAGC

ATTTGCA

>NIG9_Afunvgsc [organism=Anopheles funestus] [Nigeria] vgsc gDNA, partial CDS

GTTTAATGAAGCCCCTCAAATGGTTGATACATTCGTAAAAAGACTAGAAA

CATAATTCGAAAGACTAGAGATGAAACAATGAATAAGACTGAAGGTAGAG

TATTTAGCGTACAATATCCATTGTTTCTTAAACATATTGCTTTATGTTCA

AATATGCGCCTTGCAAATCAATTACAATTTATAACTTTTTATCATTTGTA

AGAAAATCTATGACTGCTGATGTGTCACCTAAATTATTTACAATTGTGTC

GTTATTTGTTATCTGATCATTAAACACAAAGGTTTAAGGTCGATGCATCC

GTATAAATTTGTGTAAAACTACAATAATTTCATTTGAGTGCCAGAGACTT

CAACGATCGTATTTAAATCAGTCGCTATTTTCTTGCATTTGGTATTTTGT

TCTGATTTACAGCGAACATGTACTAGACTGTTCAATGTCCGCTGGTTACT

GTCTTGCATACCATTGTTTGCTAAACAAAACAAAATAACTACAAATTAAA

CGACAAGCTTTTAGAACAACAAGAGCTCTAGAAACAGCTTACATATGCCT

CGATAATCTGCAATCTGCTAGCTTCTCCACGCGGATTATTTGTAAATCAA

GTGCATTCACATTTTTCTTAGAGTGCTGGTGAACGCCTAATCCTAATCGC

GCATCGTTCGTATTTTTCTTCAATTCTATTCGAAATAAATTCTTCCAGAT

AATGTGCATCTGTTCCCAGACCAAGATCTGCCAAGATGGAATTTTACCGA

TTTCATGCATTCCTTTATGATTGTGTTCCGTGTGCTGTGCGGTGAATGGA

TCGAATCCATGTGGGACTGTATGCTTGTTGGCGATGTGTCATGCATACCA

TTTTTCCTGGCTACGGTAGTAATAGGAAATTTAGTCGTAAGTAACCTAGC

ATTTGCA

>NIG10_Afunvgsc [organism=Anopheles funestus] [Nigeria] vgsc gDNA, partial CDS

GTTCAATGAAGCCCCTCAAATGGTTGATACATTCGTAAAAAGACTAGAAA

CATAATTCGAAAGACTAGAGATGAAACAATGAATAAGACTGAAGGTAGAG

TATTTAGCGTACAATATCCATTGTTTCTTAAACATATTGCTTTATGTTCA

AATATGCGCCTTGCAAATCAATTACAATTTATAACTTTTTATCATTTGTA

AGAAAATCTATGACTGCTGATGTGTCACCTAAATTATTTACAATTGTGTC

GTTATTTGTTATCTGATCACTAAACACAAAGGTTTAAGGTCGATGCATCC

GTATAAATTTGTGTAAAACTACAATAATTTCATTTGAGTGCCAGAGACTT

CAACGATCGTATTTAAATCAGTCGCTATTTTCTTGCATTTGGTATTTTGT

TCTGATTTACAGCGAACATGTACTAGACTGTTCAATGTCCGCTGGTTACT

GTCTTGCATACCATTGTTTGCTAAACAAAACAAAATAACTACAAATTAAA

CGACAAGCTTTTAGAACAACAAGAGCTCTAGAAACAGCTTACATATGCCT

CGATAATCTGCAATCTGCTAGCTTCTCCACGCGGATTATTTGTAAATCAA

GTGCATTCACATTTTTCTTAGAGTGCTGGTGAACGCCTAATCCTAATCGC

GCATCGTTCGTATTTTTCTTCAATTCTATTCGAAATAAATTCTTCCAGAT

AATGTGCATCTGTTCCCAGACCAAGATCTGCCAAGATGGAATTTTACCGA

TTTCATGCATTCCTTTATGATTGTGTTCCGTGTGCTGTGCGGTGAATGGA

TCGAATCCATGTGGGACTGTATGCTTGTTGGCGATGTGTCATGCATACCA

TTTTTCCTGGCTACGGTAGTAATAGGAAATTTAGTCGTAAGTAACCTAGC

ATTTGCA

>NIG11_Afunvgsc [organism=Anopheles funestus] [Nigeria] vgsc gDNA, partial CDS

GTTCAATGAAGCCCCTCAAATGGTTGATACATTCGTAAAAAAGATAGAAA

CATAATTCGAAAGACTAGAGATGAAACAATGAATAAGACTGAAGGTAGAG

TATTTAGCGTACAATATCCATTGTTTCTTAAACATATTGCTTTATGTTCA

AATATGCGCCTTGCAAATCAATTACAATTTATAACTTTTTATCATTTGTA

AGAAAATCTATGACTGCTGATGTGTCACCTAAATTATTTACAATTGTGTC

GTTATTTGTTATCTGATCACTAAACACAAAGGTTTAAGGTCGATGCATCC

GTATAAATTTGTGTAAAACTACAATAATTTCATTTGAGTGCCAGAGACTT

CAACGATCGTATTTAAATCAGTCGCTATTTTCTTGCATTTGGTATTTTGT

TCTGATTTACAGCGAACATGTACTAGACTGTTCAATGTCCGCTGGTTACT

GTCTTGCATACCATTGTTTGCTAAACAAAACAAAATAACTACAAATTAAA

CGACAAGCTTTTAGAACAACAAGAGCTCTAGAAACAGCTTACATATGCCT

CGATAATCTGCAATCTGCTAGCTTCTCCACGCGGATTATTTGTAAATCAA

GTGCATTCACATTTTTCTTAGAGTGCTGGTGAACGCCTAATCCTAATCGC

GCATCGTTCGTATTTTTCTTCAATTCTATTCGAAATAAATTCTTCCAGAT

AATGTGCATCTGTTCCCAGACCAAGATCTGCCAAGATGGAATTTTACCGA

TTTCATGCATTCCTTTATGATTGTGTTCCGTGTGCTGTGCGGTGAATGGA

TCGAATCCATGTGGGACTGTATGCTTGTTGGCGATGTGTCATGCATACCA

TTTTTCCTGGCTACGGTAGTAATAGGAAATTTAGTCGTAAGTAACCTAGC

ATTTGCA

>NIG12_Afunvgsc [organism=Anopheles funestus] [Nigeria] vgsc gDNA, partial CDS

GTTCAATGAAGCCCCTCAAATGGTTGATACATTCGTAAAAAGGCTAGAAA

CCTAATTCGAAAGGCTAGAGATGAAACAATGAATAAGACTGAAGGTAGAG

TATTTAGCGTACAATATCCATTGTTTCTTAAACATATTGCTTTATGTTCA

AATATGCGCCTTGCAAATCAATTACAATTTATAACTTTTTATCATTTGTA

AGAAAATCTATGACTGCTGATGTGTCACCTAAATTATTTACAATTGTGTC

GTTATTTGTTATCTGATCACTAAACACAAAGGTTTAAGGTCGATGCATCC

GTATAAATTTGTGTAAAACTACAATAATTTCATTTGAGTGCCAGAGACTT

CAACGATCGTATTTAAATCAGTCGCTATTTTCTTGCATTTGGTATTTTGT

TCTGATTTACAGCGAACATGTACTAGACTGTTCAATGTCCGCTGGTTACT

GTCTTGCATACCATTGTTTGCTAAACAAAACAAAATAACAACAAATTAAA

CGACAAGCTTTTACAACAACAAGAGCTCTAGAAACAGCTTACATATGCCT

CGATAATCTGCAATCTGCTAGCTTCTCCACGCGGATTATTTGTAAATCAA

GTGCATTCACATTTTTCTTAGAGTGCTGGTGAACGCCTAATCCTAATCGC

GCATCGTTCGTATTTTTCTTCAATTCTATTCGAAATAAATTCTTCCAGAT

AATGTGCATCTGTTCCCAGACCAAGATCTGCCAAGATGGAATTTTACCGA

TTTCATGCATTCCTTTATGATTGTGTTCCGTGTGCTGTGCGGTGAATGGA

TCGAATCCATGTGGGACTGTATGCTTGTTGGCGATGTGTCATGCATACCA

TTTTTCCTGGCTACGGTAGTAATAGGAAATTTAGTCGTAAGTAACCTAGC

ATTTGCA

>NIG13_Afunvgsc [organism=Anopheles funestus] [Nigeria] vgsc gDNA, partial CDS

GTTCAATGAAGCCCCTCAAATGGTTGATACATTCGTAAAAAGACTAGAAA

CATAATTCGAAAGACTAGAGATGAAACAATGAATAAGACTGAAGGTAGAG

TATTTAGCGTACAATATCCATTGTTTCTTAAACATATTGCTTTATGTTCA

AATATGCGCCTTGCAAATCAATTACAATTTATAACTTTTTATCATTTGTA

AGAAAATCTATGACTGCTGATGTGTCACCTAAATTATTTACAATTCTGTC

GTTATTTGTTATCTGATCACTAAACACAAAGGTTTAAGGTCGATGCATCC

GTATAAATTTGTGTAAAACTACAATAATTTCATTTGAGTGCCAGAGACTT

CAACGATCGTATTTAAATCAGTCGCTATTTTCTTGCATTTGGTATTTTGT

TCTGATTTACAGCGAACATGTACTAGACTGTTCAATGTCCGCTGGTTACT

GTCTTGCATACCATTGTTTGCTAAACAAAACAAAATAACAACAAATTAAA

CGACAAGCTTTTAGAACAACAAGAGCTCTAGAAACAGCTTACATATGCCT

CGATAATCTGCAATCTGCTAGCTTCTCCACGCGGATTATTTGTAAATCAA

GTGCATTCACATTTTTCTTAGAGTGCTGGTGAACGCCTAATCCTAATCGC

GCATCGTTCGTATTTTTCTTCAATTCTATTCGAAATAAATTCTTCCAGAT

AATGTGCATCTGTTCCCAGACCAAGATCTGCCAAGATGGAATTTTACCGA

TTTCATGCATTCCTTTATGATTGTGTTCCGTGTGCTGTGCGGTGAATGGA

TCGAATCCATGTGGGACTGTATGCTTGTTGGCGATGTGTCATGCATACCA

TTTTTCCTGGCTACGGTAGTAATAGGAAATTTAGTCGTAAGTAACCTAGC

ATTTGCA

>NIG14_Afunvgsc [organism=Anopheles funestus] [Nigeria] vgsc gDNA, partial CDS

GTTTAATAAAGCCCCTCAAATGGTTGATACATTCGTAAAAAGACTAGAAA

CATAATTCGAAAGACTAGAGATGAAACAATGAATAAGACTGAAGGTAGAG

TATTTAGCGTACAATATCCATTGTTTCTTAAACATATTGCTTTATGTTCA

AATATGCGCCTTGCAAATCAATTACAATTTATAACTTTTTATCATTTGTA

AGAAAATCTATGACTGCTGATGTGTCACCTAAATTATTTACAATTGTGTC

GTTATTTGTTATCTGATCACTAAACACAAAGGTTTAAGGTCGATGCATCC

GTATAAATTTGTGTAAAACTACAATAATTTCATTTGAGTGCCAGAGACTT

CAACGATCGTATTTAAATCAGTCGCTATTTTCTTGCATTTGGTATTTTGT

TCTGATTTACAGCGAACATGTACTAGACTGTTCAATGTCCGCTGGTTACT

GTCTTGCATACCATTGTTTGCTAAACAAAACAAAATAACAACAAATTAAA

CGACAAGCTTTTAGAACAACAAGAGCTCTAGAAACAGCTTACATATGCCT

CGATAATCTGCAATCTGCTAGCTTCTCCACGCGGATTATTTGTAAATCAA

GTGCATTCACATTTTTCTCAGAGTGCTGGTGAACGCCTAATTCTAATCGC

GCATCGTTCGTATTTTTCTTCAATTCTATTCGAAATAAATTCTTCCAGAT

AATGTGCATCTGTTCCCAGACCAAGATCTGCCAAGATGGAATTTTACCGA

TTTCATGCATTCCTTTATGATTGTGTTCCGTGTGCTGTGCGGTGAATGGA

TCGAATCCATGTGGGACTGTATGCTTGTTGGCGATGTGTCATGCATACCA

TTTTTCCTGGCTACGGTAGTAATAGGAAATTTAGTCGTAAGTAACCTAGC

ATTTGCA

>NIG15_Afunvgsc [organism=Anopheles funestus] [Nigeria] vgsc gDNA, partial CDS

GTTCAAAGGAACCCTTCAAATGGTTGATACATTCGTAAAAAGACTAGAAA

CATAATTCGAAAGACTAGAGATGAAACAATGAATAAGACTGAAGGTAGAG

TATTTAGCGTACAATATCCATTGTTTCTTAAACATATTGCTTTATGTTCA

AATATGCGCCTTGCAAATCAATTACAATTTATAACTTTTTATCATTTGTA

AGAAAATCTATGACTGCTGATGTGTCACCTAAATTATTTACAATTGTGTC

GTTATTTGTTATCTGATCATTAAACACAAAGGTTTAAGGTCGATGCATCC

GTATAAATTTGTGTAAAACTACAATAATTTCATTTGAGTGCCAGAGACTT

CAACGATCGTATTTAAATCAGTCGCTATTTTCTTGCATTTGGTATTTTGT

TCTGATTTACAGCGAACATGTACTAGACTGTTCAATGTCCGCTGGTTACT

GTCTTGCATACCATTGTTTGCTAAACAAAACAAAATAACAACAAATTAAA

CGACAAGCTTTTAGAACAACAAGAGCTCTAGAAACAGCTTACATATGCCT

CGATAATCTGCAATCTGCTAGCTTCTCCACGCGGATTATTTGTAAATCAA

GTGCATTCACATTGTTCTCAGAGTGCTGGTGAACGCCTAATCCTAATCGC

GCATCGTTCGTATTTTTCTTCAATTCTATTCGAAATAAATTCTTCCAGAT

AATGTGCATCTGTTCCCAGACCAAGATCTGCCAAGATGGAATTTTACCGA

TTTCATGCATTCCTTTATGATTGTGTTCCGTGTGCTGTGCGGTGAATGGA

TCGAATCCATGTGGGACTGTATGCTTGTTGGCGATGTGTCATGCATACCA

TTTTTCCTGGCTACGGTAGTAATAGGAAATTTAGTCGTAAGTAACCTAGC

ATTTGCA

>NIG16_Afunvgsc [organism=Anopheles funestus] [Nigeria] vgsc gDNA, partial CDS

GTTCAAAGGAACCCTTCAAATGGTTGATACATTCGTAAAAAGACTAGAAA

CATAATTCGAAAGACTAGAGATGAAACAATGAATAAGACTGAAGGTAGAG

TATTTAGCGTACAATATCCATTGTTTCTTAAACATATTGCTTTATGTTCA

AATATGCGCCTTGCAAATCAATTACAATTTATAACTTTTTATCATTTGTA

AGAAAATCTATGACTGCTGATGTGTCACCTAAATTATTTACAATTGTGTC

GTTATTTGTTATCTGATCATTAAACACAAAGGTTTAAGGTCGATGCATCC

GTATAAATTTGTGTAAAACTACAATAATTTCATTTGAGTGCCAGAGACTT

CAACGATCGTATTTAAATCAGTCGCTATTTTCTTGCATTTGGTATTTTGT

TCTGATTTACAGCGAACATGTACTAGACTGTTCAATGTCCGCTGGTTACT

GTCTTGCATACCATTGTTTGCTAAACAAAACAAAATAACAACAAATTAAA

CGACAAGCTTTTAGAACAACAAGAGCTCTAGAAACAGCTTACATATGCCT

CGATAATCTGCAATCTGCTAGCTTCTCCACGCGGATTATTTGTAAATCAA

GTGCATTCACATTGTTCTCAGAGTGCTGGTGAACGCCTAATCCTAATCGC

GCATCGTTCGTATTTTTCTTCAATTCTATTCGAAATAAATTCTTCCAGAT

AATGTGCATCTGTTCCCAGACCAAGATCTGCCAAGATGGAATTTTACCGA

TTTCATGCATTCCTTTATGATTGTGTTCCGTGTGCTGTGCGGTGAATGGA

TCGAATCCATGTGGGACTGTATGCTTGTTGGCGATGTGTCATGCATACCA

TTTTTCCTGGCTACGGTAGTAATAGGAAATTTAGTCGTAAGTAACCTAGC

ATTTGCA

>NIG17_Afunvgsc [organism=Anopheles funestus] [Nigeria] vgsc gDNA, partial CDS

GTTCAATGAAGCCCCTCAAATGGTTGATACATTCGTAAAAAGACTAGAAA

CATAATTCGAAAGACTAGAGATGAAACAATGAATAAGACTGAAGGTAGAG

TATTTAGCGTACAATATCCATTGTTTCTTAAACATATTGCTTTATGTTCA

AATATGCGCCTTGCAAATCAATTACAATTTATAACTTTTTATCATTTGTA

AGAAAATCTATGACTGCTGATGTGTCACCTAAATTATTTACAATTGTGTC

GTTATTTGTTATCTGATCACTAAACACAAAGGTTTAAGGTCGATGCATCC

GTATAAATTTGTGTAAAACTACAATAATTTCATTTGAGTGCCAGAGACTT

CAACGATCGTATTTAAATCAGTCGCTATTTTCTTGCATTTGGTATTTTGT

TCTGATTTACAGCAAACATGTACTAGACTGTTCAATGTCCGCTGGTTACT

GTCTTGCATACCATTGTTTGCTAAACAAAACAAAATAACAACAAATTAAA

CGACAAGCTTTTAGAACAACAAGAGCTCTAGAAACAGCTTACATATGCCT

CGATAATCTGCAATCTGCTAGCTTCTCCACGCGGATTATTTGTAAATCAA

GTGCATTCACATTTTTCTTAGAGTGCTGGTGAACGCCTAATCCTAATCGC

GCATCGTTCGTATTTTTCTTCAATTCTATTCGAAATAAATTCTTCCAGAT

AATGTGCATCTGTTCCCAGACCAAGATCTGCCAAGATGGAATTTTACCGA

TTTCATGCATTCCTTTATGATTGTGTTCCGTGTGCTGTGCGGTGAATGGA

TCGAATCCATGTGGGACTGTATGCTTGTTGGCGATGTGTCATGCATACCA

TTTTTCCTGGCTACGGTAGTAATAGGAAATTTAGTCGTAAGTAACCTAGC

ATTTGCA

>NIG18_Afunvgsc [organism=Anopheles funestus] [Nigeria] vgsc gDNA, partial CDS

GTTCAATGAAGCCCCTCAAATGGTTGATACATTCGTAAAAAGACTAGAAA

CATAATTCGAAAGACTAGAGATGAAACAATGAATAAGACTGAAGGTAGAG

TATTTAGCGTACAATATCCATTGTTTCTTAAACATATTGCTTTATGTTCA

AATATGCGCCTTGCAAATCAATTACAATTTATAACTTTTTATCATTTGTA

AGAAAATCTATGACTGCTGATGTGTCACCTAAATTATTTACAATTGTGTC

GTTATTTGTTATCTGATCACTAAACACAAAGGTTTAAGGTCGATGCATCC

GTATAAATTTGTGTAAAACTACAATAATTTCATTTGAGTGCCAGAGACTT

CAACGATCGTATTTAAATCAGTCGCTATTTTCTTGCATTTGGTATTTTGT

TCTGATTTACAGCGAACATGTACTAGACTGTTCAATGTCCGCTGGTTACT

GTCTTGCATACCATTGTTTGCTAAACAAAACAAAATAACTACAAATTAAA

CGACAAGCTTTTAGAACAACAAGAGCTCTAGAAACAGCTTACATATGCCT

CGATAATCTGCAATCTGCTAGCTTCTCCACGCGGATTATTTGTAAATCAA

GTGCATTCACATTTTTCTTAGAGTGCTGGTGAACGCCTAATCCTAATCGC

GCATCGTTCGTATTTTTCTTCAATTCTATTCGAAATAAATTCTTCCAGAT

AATGTGCATCTGTTCCCAGACCAAGATCTGCCAAGATGGAATTTTACCGA

TTTCATGCATTCCTTTATGATTGTGTTCCGTGTGCTGTGCGGTGAATGGA

TCGAATCCATGTGGGACTGTATGCTTGTTGGCGATGTGTCATGCATACCA

TTTTTCCTGGCTACGGTAGTAATAGGAAATTTAGTCGTAAGTAACCTAGC

ATTTGCA

>NIG19_Afunvgsc [organism=Anopheles funestus] [Nigeria] vgsc gDNA, partial CDS

GTTTAATAAAGCCCCTCAAATGGTTGATACATTCGTAAAAAGACTAGAAA

CATAATTCGAAAGACTAGAGATGAAACAATGAATAAGACTGAAGGTAGAG

TATTTAGCGTACAATATCCATTGTTTCTTAAACATATTGCTTTATGTTCA

AATATGCGCCTTGCAAATCTATTACAATTTATAACTTTTTATCATTTGTA

AGAAAATCTATGACTGCTGATGTGTCACCTAAATTATTTACAATTGTGTC

GTTATTTGTTATCTGATCACTAAACACAAAGGTTTAAGGTCGATGCATCC

GTATAAATTTGTGTAAAACTACAATAATTTCATTTGAGTGCCAGAGACTT

CAACGATCGTATTTAAATCAGTCGCTATTTTCTTGCATTTGGTATTTTGT

TCTGATTTACAGCGAACATGTACTAGACTGTTCAATGTCCGCTGGTTACT

GTCTTGCATACCATTGTTTGCTAAACAAAACAAAATAACTACAAATTAAA

CGACAAGCTTTTAGAACAACAAGAGCTCTAGAAACAGCTTACATATGCCT

CGATAATCTGCAATCTGCTAGCTTCTCCACGCGGATTATTTGTAAATCAA

GTGCATTCACATTTTTCTCAGAGTGCTGGTGAACGCCTAATCCTAATCGC

GCATCGTTCGTATTTTTCTTCAATTCTATTCGAAATAAATTCTTCCAGAT

AATGTGCATCTGTTCCCAGACCAAGATCTGCCAAGATGGAATTTTACCGA

TTTCATGCATTCCTTTATGATTGTGTTCCGTGTGCTGTGCGGTGAATGGA

TCGAATCCATGTGGGACTGTATGCTTGTTGGCGATGTGTCATGCATACCA

TTTTTCCTGGCTACGGTAGTAATAGGAAATTTAGTCGTAAGTAACCTAGC

ATTTGCA

>NIG20_Afunvgsc [organism=Anopheles funestus] [Nigeria] vgsc gDNA, partial CDS

GTTCAATGAAGCCCCTCAAATGGTTGATACATTCGTAAAAAGACTAGAAA

CATAATTCGAAAGACTAGAGATGAAACAATGAATAAGACTGAAGGTAGAG

TATTTAGCGTACAATATCCATTGTTTCTTAAACATATTGCTTTATGTTCA

AATATGCGCCTTGCAAATCAATTACAATTTATAACTTTTTATCATTTGTA

AGAAAATCTATGACTGCTGATGTGTCACCTAAATTATTTACAATTGTGTC

GTTATTTGTTATCTGATCACTAAACACAAAGGTTTAAGGTCGATGCATCC

GTATAAATTTGTGTAAAACTACAATAATTTCATTTGAGTGCCAGAGACTT

CAACGATCGTATTTAAATCAGTCGCTATTTTCTTGCATTTGGTATTTTGT

TCTGATTTACAGCGAACATGTACTAGACTGTTCAATGTCCGCTGGTTACT

GTCTTGCATACCATTGTTTGCTAAACAAAACAAAATAACAACAAATTAAA

CGACAAGCTTTTAGAACAACAAGAGCTCTAGAAACAGCTTACATATGCCT

CGATAATCTGCAATCTGCTAGCTTCTCCACGCGGATTATTTGTAAATCAA

GTGCATTCACATTTTTCTTAGAGTGCTGGTGAACGCCTAATCCTAATCGC

GCATCGTTCGTATTTTTCTTCAATTCTATTCGAAATAAATTCTTCCAGAT

AATGTGCATCTGTTCCCAGACCAAGATCTGCCAAGATGGAATTTTACCGA

TTTCATGCATTCCTTTATGATTGTGTTCCGTGTGCTGTGCGGTGAATGGA

TCGAATCCATGTGGGACTGTATGCTTGTTGGCGATGTGTCATGCATACCA

TTTTTCCTGGCTACGGTAGTAATAGGAAATTTAGTCGTAAGTAACCTAGC

ATTTGCA

>NIG21_Afunvgsc [organism=Anopheles funestus] [Nigeria] vgsc gDNA, partial CDS

GTTCAATGAAGCCCCTCAAATGGTTGATACATTCGTAAAAAGACTAGAAA

CATAATTCGAAAGACTAGAGATGAAACAATGATTAAGACTGAAGGTAGAG

TATTTAGCGTACAATATCCATTGTTTCTTAAACATATTGCTTTATGTTCA

AATATGCGCCTTGCAAATCAATTACAATTTATAACTTTTTATCATTTGTA

AGAAAATCTATGACTGCTAATGCGTCACCTAAATTATTTACAATTGTGTC

GTTATTTGTTATCTGATCACTAAACACAAAGGTTTAAGGTCGATGCATCC

GTATAAATTTGTGTAAAACTACAATAATTTCATTTGAGTGCCAGAGACTT

CAACGATCGTATTTAAATCAGTCGCTATTTTCTTGCATTTGGTATTTTGT

TCTGATTTACAGCGAACATGTACTAGACTGTTCAATGTCCGCTGGTTACT

GTCTTGCATACCATTGTTTGCTAAACAAAACAAAATAACAACAAATTAAA

CGACAAGCTTTTAGAACAACAAGAGCTCTAGAAACAGCTTACATATGCCT

CGATAATCTGCAATCTGCTAGCTTCTCCACGCGGATTATTTGTAAATCAA

GTGCATTCACATTTTTGTCAGAGTGCTGGTGAACGCCTAATCCTAATCGC

GCATCGTTCGTATTTTTCTTCAATTCTATTCGAAATAAATTCTTCCAGAT

AATGTGCATCTGTTCCCAGACCAAGATCTGCCAAGATGGAATTTTACCGA

TTTCATGCATTCCTTTATGATTGTGTTCCGTGTGCTGTGCGGTGAATGGA

TCGAATCCATGTGGGACTGTATGCTTGTTGGCGATGTGTCATGCATACCA

TTTTTCCTGGCTACGGTAGTAATAGGAAATTTAGTCGTAAGTAACCTAGC

ATTTGCA

>NIG22_Afunvgsc [organism=Anopheles funestus] [Nigeria] vgsc gDNA, partial CDS

GTTTAATGAAGCCCCTCAAATGGTTGATACATTCGTAAAAAGACTAGAAA

CATAATTCGAAAGACTAGAGATGAAACAATGAATAAGACTGAAGGTAGAG

TATTTAGCGTACAATATCCATTGTTTCTTAAACATATTGCTTTATGTTCA

AATATGCGCCTTGCAAATCAATTACAATTTATAACTTTTTATCATTTGTA

AGAAAATCTATGACTGCTGATGTGTCACCTAAATTATTTACAATTGTGTC

GTTATTTGTTATCTGATCACTAAACACAAAGGTTTAAGGTCGATGCATCC

GTATAAATTTGTGTAAAACTACAATAATTTCATTTGAGTGCCAGAGACTT

CAACGATCGTATTTAAATCAGTCGCTATTTTCTTGCATTTGGTATTTTGT

TCTGATTTACAGCGAACATGTACTAGACTGTTCAATGTCCGCTGGTTACT

GTCTTGCATACCATTGTTTGCTAAACAAAACAAAATAACAACAAATTAAA

CGACAAGCTTTTAGAACAACAAGAGCTCTAGAAACAGCTTACATATGCCT

CGATAATCTGCAATCTGCTAGCTTCTCCACGCGGATTATTTGTAAATCAA

GTGCATTCACATTTTTCTCAGAGTGCTGGTGAACGCCTAATCCTAATCGC

GCATCGTTCGTATTTTTCTTCAATTCTATTCGAAATAAATTCTTCCAGAT

AATGTGCATCTGTTCCCAGACCAAGATCTGCCAAGATGGAATTTTACCGA

TTTCATGCATTCCTTTATGATTGTGTTCCGTGTGCTGTGCGGTGAATGGA

TCGAATCCATGTGGGACTGTATGCTTGTTGGCGATGTGTCATGCATACCA

TTTTTCCTGGCTACGGTAGTAATAGGAAATTTAGTCGTAAGTAACCTAGC

ATTTGCA

>NIG23_Afunvgsc [organism=Anopheles funestus] [Nigeria] vgsc gDNA, partial CDS

GTTCAATGAAGCCCCTCAAATGGTTGATACATTCGTAAAAAAGCTAGAAA

CATAATTCGAAAGACTAGAGATGAAACAATGAATAAGACTGAAGGTAGAG

TATTTAGCGTACAATATCCATTGTTTCTTAAACATATTGCTTTATGTTCA

AATATGCGCCTTGCAAATCAATTACAATTTATAACTTTTTATCATTTGTA

AGAAAATCTATGACTGCTAATGTGTCACCTAAATTATTTACAATTGTGTC

GTTATTTGTTATCTGATCACTAAACACAAAGGTTTAAGGTCGATGCATCC

GTATAAATTTGTGTAAAACTACAATAATTTCATTTGAGTGCCAGAGACTT

CAACGATCGTATTTAAATCAGTCGCTATTTTCTTGCATTTGGTATTTTGT

TCTGATTTACAGCGAACATGTACTAGACTGTTCAATGTCCGCTGGTTACT

GTCTTGCATACCATTGTTTGATAAACAAAACAAAATAACAACAAATTAAA

CGACAAGCTTTTAGAACAACAAGAGCTCTAGAAACAGCTTACATATGCCT

CGATAATCTGCAATCTGCTAGCTTCTCCACGCGGATTATTTGTAAATCAA

GTGCATTCACATTTTTCTTAGAGTGCTGGTGAACGCCTAATCCTAATCGC

GCATCGTTCGTATTTTTCTTCAATTCTATTCGAAATAAATTCTTCCAGAT

AATGTGCATCTGTTCCCAGACCAAGATCTGCCAAGATGGAATTTTACCGA

TTTCATGCATTCCTTTATGATTGTGTTCCGTGTGCTGTGCGGTGAATGGA

TCGAATCCATGTGGGACTGTATGCTTGTTGGCGATGTGTCATGCATACCA

TTTTTCCTGGCTACGGTAGTAATAGGAAATTTAGTCGTAAGTAACCTAGC

ATTTGCA

>NIG24_Afunvgsc [organism=Anopheles funestus] [Nigeria] vgsc gDNA, partial CDS

GTTTAATAAAGCCCCTCAAATGGTTGATACATTCGTAAAAAGACTAGAAA

CATAATTCGAAAGACTAGAGATGAAACAATGAATAAGACTGAAGGTAGAG

TATTTAGCGTACAATATCCATTGTTTCTTAAACATATTGCTTTATGTTCA

AATATGCGCCTTGCAAATCAATTACAATTTATAACTTTTTATCATTTGTA

AGAAAATCTATGACTGCTGATGTGTCACCTAAATTATTTACAATTGTGTC

GTTATTTGTTATCTGATCACTAAACACAAAGGTTTAAGGTCGATGCATCC

GTATAAATTTGTGTAAAACTACAATAATTTCATTTGAGTGCCAGAGACTT

CAACGATCGTATTTAAATCAGTCGCTATTTTCTTGCATTTGGTATTTTGT

TCTGATTTACAGCGAACATGTACTAGACTGTTCAATGTCCGCTGGTTACT

GTCTTGCATACCATTGTTTGCTAAACAAAACAAAATAACAACAAATTAAA

CGACAAGCTTTTAGAACAACAAGAGCTCTAGAAACAGCTTACATATGCCT

CGATAATCTGCAATCTGCTAGCTTCTCCACGCGGATTATTTGTAAATCAA

GTGCATTCACATTTTTCTTAGAGTGCTGGTGAACGCCTAATCCTAATCGC

GCATCGTTCGTATTTTTCTTCAATTCTATTCGAAATAAATTCTTCCAGAT

AATGTGCATCTGTTCCCAGACCAAGATCTGCCAAGATGGAATTTTACCGA

TTTCATGCATTCCTTTATGATTGTGTTCCGTGTGCTGTGCGGTGAATGGA

TCGAATCCATGTGGGACTGTATGCTTGTTGGCGATGTGTCATGCATACCA

TTTTTCCTGGCTACGGTAGTAATAGGAAATTTAGTCGTAAGTAACCTAGC

ATTTGCA

>NIG25_Afunvgsc [organism=Anopheles funestus] [Nigeria] vgsc gDNA, partial CDS

GTTTAATGAAGCCCCTCAAATGGTTGATACATTCGTAAAAAGACTAGAAA

CATAATTCGAAAGACTAGAGATGAAACAATGAATAAGACTGAAGGTAGAG

TATTTAGCGTACAATATCCATTGTTTCTTAAACATATTGCTTTATGTTCA

AATATGCGCCTTGCAAATCAATTACAATTTATAACTTTTTATCATTTGTA

AGAAAATCTATGACTGCTGATGTGTCACCTAAATTATTTACAATTGTGTC

GTTATTTGTTATCTGATCACTAAACACAAAGGTTTAAGGTCGATGCATCC

GTATAAATTTGTGTAAAACTACAATAATTTCATTTGAGTGCCAGAGACTT

CAACGATCGTATTTAAATCAGTCGCTATTTTCTTGCATTTGGTATTTTGT

TCTGATTTACAGCGAACATGTACTAGACTGTTCAATGTCCGCTGGTTACT

GTCTTGCATACCATTGTTTGCTAAACAAAACAAAATAACAACAAATTAAA

CGACAAGCTTTTAGAACAACAAGAGCTCTAGAAACAGCTTACATATGCCT

CGATAATCTGCAATCTGCTAGCTTCTCCACGCGGATTATTTGTAAATCAA

GTGCATTCACATTTTTCTCAGAGTGCTGGTGAACGCCTAATCCTAATCGC

GCATCGTTCGTATTTTTCTTCAATTCTATTCGAAATAAATTCTTCCAGAT

AATGTGCATCTGTTCCCAGACCAAGATCTGCCAAGATGGAATTTTACCGA

TTTCATGCATTCCTTTATGATTGTGTTCCGTGTGCTGTGCGGTGAATGGA

TCGAATCCATGTGGGACTGTATGCTTGTTGGCGATGTGTCATGCATACCA

TTTTTCCTGGCTACGGTAGTAATAGGAAATTTAGTCGTAAGTAACCTAGC

ATTTGCA

>NIG26_Afunvgsc [organism=Anopheles funestus] [Nigeria] vgsc gDNA, partial CDS

GTTTAATGAAGCCCCTCAAATGGTTGATACATTCGTAAAAAGACTAGAAA

CATAATTCGAAAGACTAGAGATGAAACAATGAATAAGACTGAAGGTAGAG

TATTTAGCGTACAATATCCATTGTTTCTTAAACATATTGCTTTATGTTCA

AATATGCGCCTTGCAAATCAATTACAATTTATAACTTTTTATCATTTGTA

AGAAAATCTATGACTGCTGATGTGTCACCTAAATTATTTACAATTGTGTC

GTTATTTGTTATCTGATCACTAAACACAAAGGTTTAAGGTCGATGCATCC

GTATAAATTTGTGTAAAACTACAATAATTTCATTTGAGTGCCAGAGACTT

CAACGATCGTATTTAAATCAGTCGCTATTTTCTTGCATTTGGTATTTTGT

TCTGATTTACAGCGAACATGTACTAGACTGTTCAATGTCCGCTGGTTACT

GTCTTGCATACCATTGTTTGCTAAACAAAACAAAATAACAACAAATTAAA

CGACAAGCTTTTAGAACAACAAGAGCTCTAGAAACAGCTTACATATGCCT

CGATAATCTGCAATCTGCTAGCTTCTCCACGCGGATTATTTGTAAATCAA

GTGCATTCACATTTTTCTCAGAGTGCTGGTGAACGCCTAATCCTAATCGC

GCATCGTTCGTATTTTTCTTCAATTCTATTCGAAATAAATTCTTCCAGAT

AATGTGCATCTGTTCCCAGACCAAGATCTGCCAAGATGGAATTTTACCGA

TTTCATGCATTCCTTTATGATTGTGTTCCGTGTGCTGTGCGGTGAATGGA

TCGAATCCATGTGGGACTGTATGCTTGTTGGCGATGTGTCATGCATACCA

TTTTTCCTGGCTACGGTAGTAATAGGAAATTTAGTCGTAAGTAACCTAGC

ATTTGCA

>NIG27_Afunvgsc [organism=Anopheles funestus] [Nigeria] vgsc gDNA, partial CDS

TTTTAATGAAAACCCTCAAATGGTTGATACATTCGTAAAAAGACTAGAAA

CATAATTCGAAAGACTAGAGATGAAACAATGAATAAGACTGAAGGTAGAG

TATTTAGCGTACAATATCCATTGTTTCTTAAACATATTGCTTTATGTTCA

AATATGCGCCTTGCAAATCAATTACAATTTATAACTTTTTATCATTTGTA

AGAAAATCTATGACTGCTGATGTGTCACCTAAATTATTTACAATTGTGTC

GTTATTTGTTATCTGATCACTAAACACAAAGGTTTAAGGTCGATGCATCC

GTATAAATTTGTGTAAAACTACAATAATTTCATTTGAGTGCCAGAGACTT

CAACGATCGTATTTAAATCAGTCGCTATTTTCTTGCATTTGGTATTTTGT

TCTGATTTACAGCGAACATGTACTAGACTGTTCAATGTCCGCTGGTTACT

GTCTTGCATACCATTGTTTGCTAAACAAAACAAAATAACTACAAATTAAA

CGACAAGCTTTTAGAACAACAAGAGCTCTAGAAACAGCTTACATATGCCT

CGATAATCTGCAATCTGCTAGCTTCTCCACGCGGATTATTTGTAAATCAA

GTGCATTCACATTGTTCTTAGAGTGCTGATGAACGCCTAATCCTAATCGC

GCATCGTTCGTATTTTTCTTCAATTCTATTCGAAATAAATTCTTCCAGAT

AATGTGCATCTGTTCCCAGACCAAGATCTGCCAAGATGGAATTTTACCGA

TTTCATGCATTCCTTTATGATTGTGTTCCGTGTGCTGTGCGGTGAATGGA

TCGAATCCATGTGGGACTGTATGCTTGTTGGCGATGTGTCATGCATACCA

TTTTTCCTGGCTACGGTAGTAATAGGAAATTTAGTCGTAAGTAACCTAGC

ATTTGCA

>NIG28_Afunvgsc [organism=Anopheles funestus] [Nigeria] vgsc gDNA, partial CDS

ATTTTGTGAAAGCCCTCAAATGGTTGATACATTCGTAAAAAGACTAGAAA

CATAATTCGAAAGACTAGAGATGAAACAATGAATAAGACTGAAGGTAGAG

TATTTAGCGTACAATATCCATTGTTTCTTAAACATATTGCTTTATGTTCA

AATATGCGCCTTGCAAATCAATTACAATTTATAACTTTTTATCATTTGTA

AGAAAATCTATGACTGCTGATGTGTCACCTAAATTATTTACAATTGTGTC

GTTATTTGTTATCTGATCCCTAAACACAAAGGTTTAAGGTCGATGCATCC

GTATAAATTTGTGTAAAACTACAATAATTTCATTTGAGTGCCAGAGACTT

CAACGATCGTATTTAAATCAGTCGCTATTTTCTTGCATTTGGTATTTTGT

TCTGATTTACAGCGAACATGTACTAGACTGTTCAATGTCCGCTGGTTACT

GTCTTGCATACCATTGTTTGCTAAACAAAACAAAATAACAACAAATTAAA

CGACAAGCTTTTAGAACAACAAGAGCTCTAGAAACAGCTTACATATGCCT

CGATAATCTGCAATCTGCTAGCTTCTCCACGCGGATTATTTGTAAATCAA

GTGCATTCACATTTTTCTTAGAGTGCTGGTGAACGCCTAATCCTAATCGC

GCATCGTTCGTATTTTTCTTCAATTCTATTCGAAATAAATTCTTCCAGAT

AATGTGCATCTGTTCCCAGACCAAGATCTGCCAAGATGGAATTTTACCGA

TTTCATGCATTCCTTTATGATTGTGTTCCGTGTGCTGTGCGGTGAATGGA

TCGAATCCATGTGGGACTGTATGCTTGTTGGCGATGTGTCATGCATACCA

TTTTTCCTGGCTACGGTAGTAATAGGAAATTTAGTCGTAAGTAACCTAGC

ATTTGCA

>NIG29_Afunvgsc [organism=Anopheles funestus] [Nigeria] vgsc gDNA, partial CDS

TTTTAATGAAAACCCTCAAAAGGTTGATACATTCGTAAAAAGACTAGAAA

CATAATTCGAAAGACTAGAGATGAAACAATGAATAAGACTGAAGGTAGAG

TATTTAGCGTACAATATCCATTGTTTCTTAAACATATTGCTTTATGTTCA

AATATGCGCCTTGCAAATCAATTACAATTTATAACTTTTTATCATTTGTA

AGAAAATCTATGACTGCTGATGTGTCACCTAAATTATTTACAATTGTGTC

GTTATTTGTTATCTGATCACTAAACACAAAGGTTTAAGGTCGATGCATCC

GTATAAATTTGTGTAAAACTACAATAATTTCATTTGAGTGCCAGAGACTT

CAACGATCGTATTTAAATCAGTCGCTATTTTCTTGCATTTGGTATTTTGT

TCTGATTTACAGCGAACATGTACTAGACTGTTCAATGTCCGCTGGTTACT

GTCTTGCATACCATTGTTTGCTAAACAAAACAAAATAACTACAAATTAAA

CGACAAGCTTTTAGAACAACAAGAGCTCTAGAAACAGCTTACATATGCCT

CGATAATCTGCAATCTGCTAGCTTCTCCACGCGGATTATTTGTAAATCAA

GTGCATTCACATTTTTCTTAGAGTGCTGGTGAACGCCTAATCCTAATCGC

GCATCGTTCGTATTTTTCTTCAATTCTATTCGAAATAAATTCTTCCAGAT

AATGTGCATCTGTTCCCAGACCAAGATCTGCCAAGATGGAATTTTACCGA

TTTCATGCATTCCTTTATGATTGTGTTCCGTGTGCTGTGCGGTGAATGGA

TCGAATCCATGTGGGACTGTATGCTTGTTGGCGATGTGTCATGCATACCA

TTTTTCCTGGCTACGGTAGTAATAGGAAATTTAGTCGTAAGTAACCTAGC

ATTTGCA

>NIG30_Afunvgsc [organism=Anopheles funestus] [Nigeria] vgsc gDNA, partial CDS

GTTTAATAAAGCCCCTCAAATGGTTGATACATTCGTAAAAAGACTAGAAA

CATAATTCGAAAGACTAGAGATGAAACAATGAATAAGACTGAAGGTAGAG

TATTTAGCGTACAATATCCATTGTTTCTTAAACATATTGCTTTATGTTCA

AATATGCGCCTTGCAAATCAATTACAATTTATAACTTTTTATCATTTGTA

AGAAAATCTATGACTGCTGATGTGTCACCTAAATTATTTACAATTGTGTC

GTTATTTGTTATCTGATCATTAAACACAAAGGTTTAAGGTCGATGCATCC

GTATAAATTTGTGTAAAACTACAATAATTTCATTTGAGTGCCAGAGACTT

CAACGATCGTATTTAAATCAGTCGCTATTTTCTTGCATTTGGTATTTTGT

TCTGATTTACAGCGAACATGTACTAGACTGTTCAATGTCCGCTGGTTACT

GTCTTGCATACCATTGTTTGCTAAACAAAACAAAATAACAACAAATTAAA

CGACAAGCTTTTAGAACAACAAGAGCTCTAGAAACAGCTTACATATGCCT

CGATAATCTGCAATCTGCTAGCTTCTCCACGCGGATTATTTGTAAATCAA

GTGCATTCACATTTTTCTCAGAGTGCTGGTGAACGCCTAATCCTAATCGC

GCATCGTTCGTATTTTTCTTCAATTCTATTCGAAATAAATTCTTCCAGAT

AATGTGCATCTGTTCCCAGACCAAGATCTGCCAAGATGGAATTTTACCGA

TTTCATGCATTCCTTTATGATTGTGTTCCGTGTGCTGTGCGGTGAATGGA

TCGAATCCATGTGGGACTGTATGCTTGTTGGCGATGTGTCATGCATACCA

TTTTTCCTGGCTACGGTAGTAATAGGAAATTTAGTCGTAAGTAACCTAGC

ATTTGCA
