# Supplementary figures and images for: Exploring the Mechanisms of Multiple Insecticide Resistance in a Highly Plasmodium-Infected Malaria Vector Anopheles funestus Sensu Stricto from Sahel of Northern Nigeria
Source: Genes (Basel). 2020 Apr 22;11(4):454. doi: 10.3390/genes11040454 (PMC7230678; doi:10.3390/genes11040454)

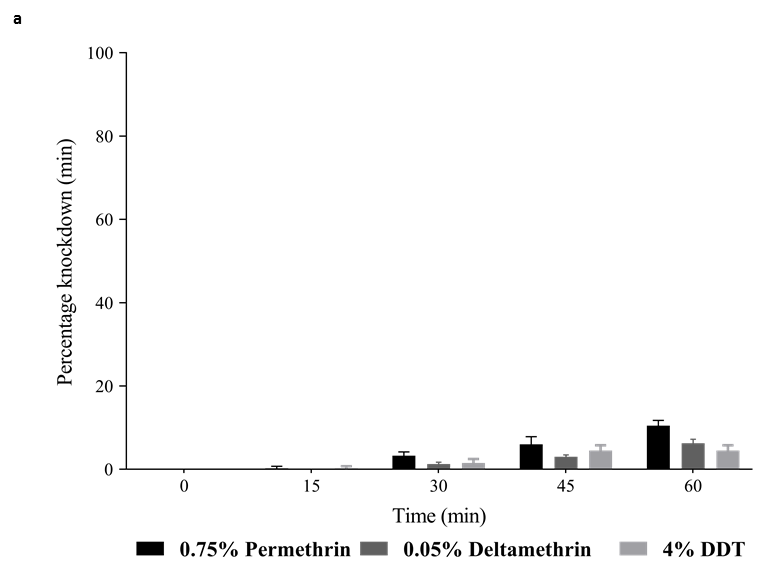

Supplement: Supplementary file 1 [file genes-11-00454-s001.zip › supplementary/Figure S1.tif]

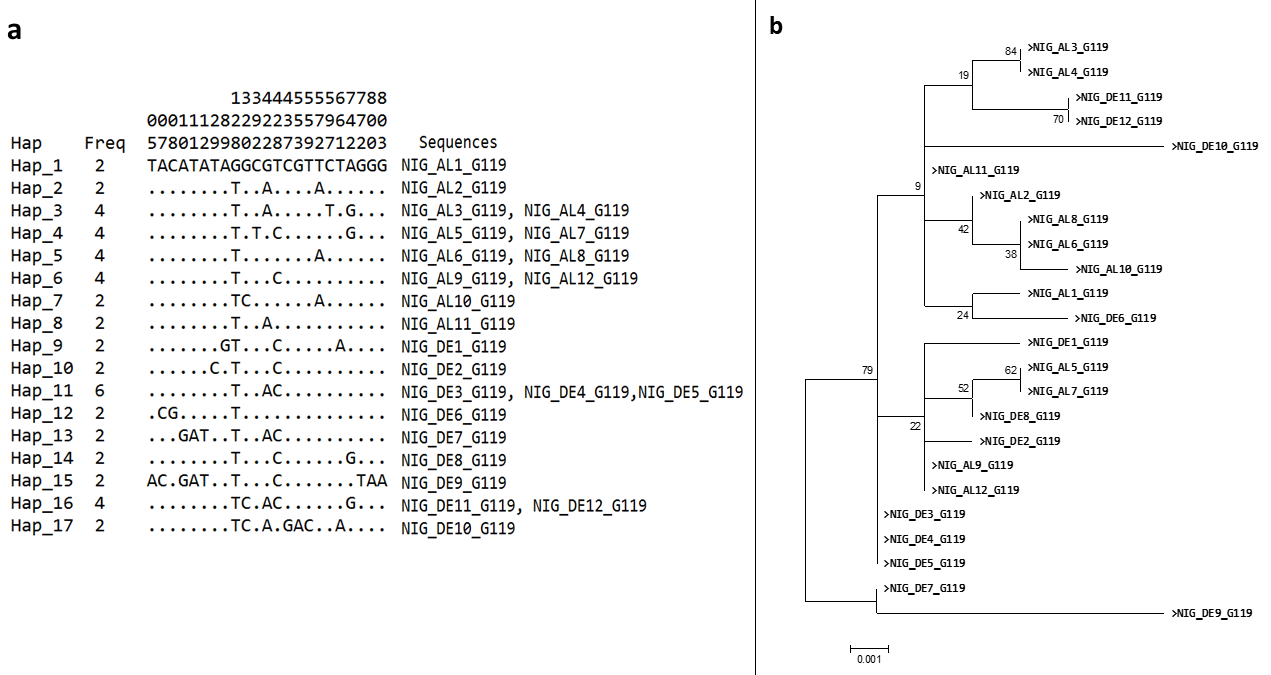

Supplement: Supplementary file 1 [file genes-11-00454-s001.zip › supplementary/Figure S2.tif]

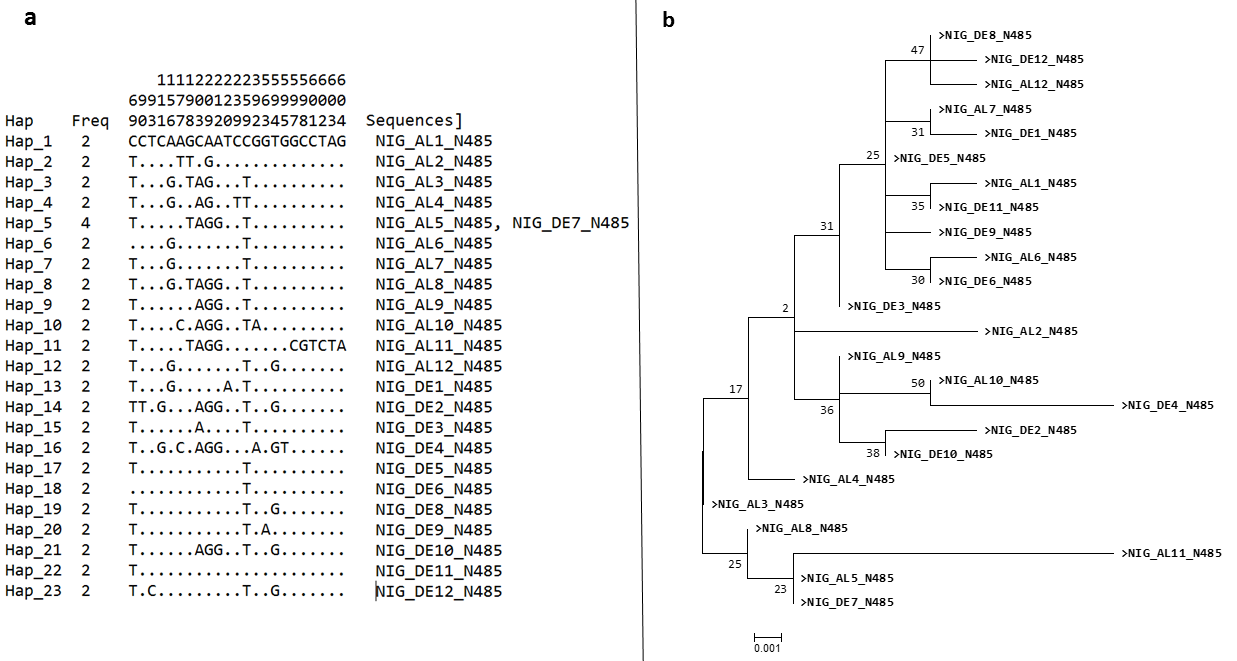

Supplement: Supplementary file 1 [file genes-11-00454-s001.zip › supplementary/Figure S3.tif]

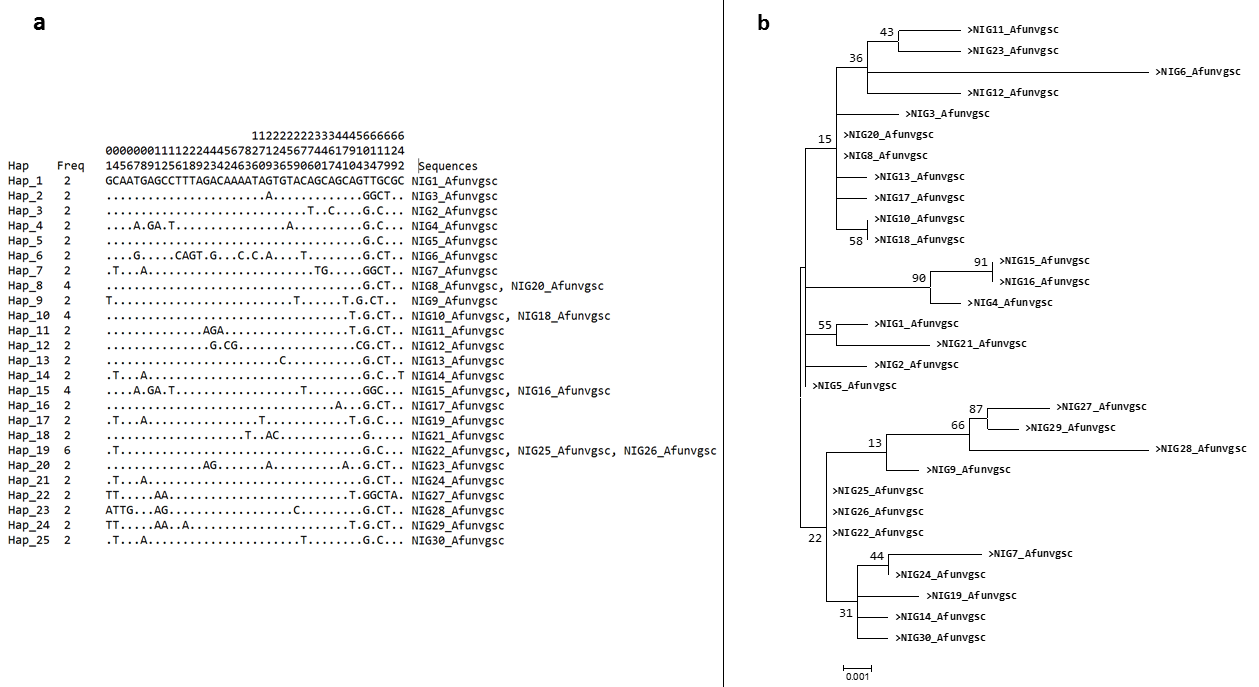

Supplement: Supplementary file 1 [file genes-11-00454-s001.zip › supplementary/Figure S4.tif]
