# Supplementary material for: Exploring the Mechanisms of Multiple Insecticide Resistance in a Highly Plasmodium-Infected Malaria Vector Anopheles funestus Sensu Stricto from Sahel of Northern Nigeria
Source: Genes (Basel). 2020 Apr 22;11(4):454. doi: 10.3390/genes11040454 (PMC7230678; doi:10.3390/genes11040454)
Supplement: Supplementary file 1 [file genes-11-00454-s001.zip › supplementary/Tables S1-S3.doc]

**Table S1:** **Summary statistics for polymorphism of a fragment of *acetylcholinesterase*-1 encompassing the 119 codon for** alive and dead mosquitoes

| **Phenotype** | **N** | **S** | **h** | **H_d_** | **Syn** | **Nonsyn** | **π (k)** | **D (Tajima)** | **D* (Fu and Li)** |
| --- | --- | --- | --- | --- | --- | --- | --- | --- | --- |
| **NIG_AL_G119** | 12 | 8 | 8 | 0.89 | 5 | 0 | 0.0034 (2.75) | 0.91 ^ns^ | 1.33 ^ns^ |
| **NIG_DE_G119** | 12 | 19 | 9 | 0.89 | 7 | 2 | 0.058 (4.62) | −0.33 ^ns^ | 1.59 ** |
| **All** | 24 | 23 | 17 | 0.95 | 8 | 2 | 0.0049 (3.98) | −0.76 ^ns^ | 1.73 ** |

*N* = number of sequences (n); S, number of polymorphic sites; h, haplotype; H_d_, haplotype diversity Syn, Synonymous mutations; Nonsyn, Non-synonymous mutations; π, nucleotide diversity (*k*= mean number of nucleotide differences); Tajima’s D and Fu and Li’s D statistics, ns, not significant; ** = *p* < 0.02.

**Table S2:** **Summary statistics for polymorphism of a fragment of *acetylcholinesterase*-1 encompassing the 485** codon for alive and dead mosquitoes

| **Phenotype** | **N** | **S** | **h** | **H_d_** | **Syn** | **Nonsyn** | **π (k)** | **D (Tajima)** | **D* (Fu and Li)** |
| --- | --- | --- | --- | --- | --- | --- | --- | --- | --- |
| **NIG_AL_N485** | 12 | 17 | 12 | 0.96 | 5 | 0 | 0.0089 (4.93) | 0.08 ^ns^ | 1.58 ** |
| **NIG_DE_N485** | 12 | 16 | 12 | 0.96 | 7 | 0 | 0.0067 (4.12) | −0.33^ns^ | 1.55 ** |
| **All** | 24 | 23 | 23 | 0.98 | 8 | 0 | 0.0078 (4.77) | −0.38^ns^ | 1.75 ** |

*N* = number of sequences (n); S, number of polymorphic sites; h, haplotype; H_d_, haplotype diversity Syn, Synonymous mutations; Nonsyn, Non-synonymous mutations; π, nucleotide diversity (*k*= mean number of nucleotide differences); Tajima’s D and Fu and Li’s D statistics, ns, not significant; ** = *p* < 0.02.

**Table S3:** **Summary statistics for polymorphism of a fragment of voltage-gated sodium channel encompassing the 1014 codon for the field F_0_ females**

|  | **N** | **S** | **h** | **H_d_** | **Syn** | **Nonsyn** | **π (k)** | **D (Tajima)** | **D* (Fu and Li)** |
| --- | --- | --- | --- | --- | --- | --- | --- | --- | --- |
| **All** | 30 | 43 | 25 | 0.97 | 0 | 0 | 0.0066 (6.06) | −1.29^ns^ | 2.007 ** |

*N* = number of sequences (n); S, number of polymorphic sites; h, haplotype; H_d_, haplotype diversity Syn, Synonymous mutations; Nonsyn, Non-synonymous mutations; π, nucleotide diversity (*k* = mean number of nucleotide differences); Tajima’s D and Fu and Li’s D statistics, ns, not significant; ** = *p* < 0.02.
